# Synergistic Bactericidal Efficacy of Combined Sanitization Strategies Against *Listeria monocytogenes*: Mechanistic Elucidation and Practical Validation in Prepared Salad

**DOI:** 10.3390/foods15183192

**Published:** 2026-09-09

**Authors:** Jinyuan Wei, Yuang Zhao, Huirong Yang, Huabing He, Mingxin Huang, Tian Han, Qingping Zhong

**Affiliations:** 1 Guangdong Provincial Key Laboratory of Food Quality and Safety, College of Food Science, South China Agricultural University, Guangzhou 510642, China; weijinyuan2021@163.com (J.W.); yanghuirong0926@163.com (H.Y.); huahe894@gmail.com (H.H.); 18026282269@163.com (M.H.); ke30258136@163.com (T.H.); 2 College of Plant Protection, South China Agricultural University, Guangzhou 510642, China; 3 Dublin International College, South China Agricultural University, Guangzhou 510642, China; 19935368217@163.com

**Keywords:** *Listeria monocytogenes*, combined sanitization method, ultrasound, slightly acidic electrolyzed water, phenyllactic acid, viable but non-culturable state

## Abstract

*L. monocytogenes* is a persistent pathogen in prepared salads that often survives through the viable but non-culturable (VBNC) state. To address this, a study evaluated a multi-hurdle sanitization strategy combining ultrasound (US), slightly acidic electrolyzed water (SAEW), and phenyllactic acid (PLA). This triple combined treatment exhibited a significant synergistic bactericidal effect, reducing *L. monocytogenes* counts below the detection limit and completely inhibiting the formation of VBNC cells. Mechanistic analysis revealed that the treatment triggered irreversible cell membrane damage, characterized by increased membrane permeability, intracellular protein leakage, and disrupted zeta potential. When applied to prepared salads, the combined method successfully extended their refrigerated shelf life (at 4 °C) to 9 days. Furthermore, this treatment effectively maintained the texture, color, and odor profile of the samples while preserving the activities of antioxidant enzymes. Overall, the US + SAEW + PLA strategy provides a highly efficient, non-thermal approach to ensuring both the microbial safety and sensory quality of prepared salads, offering a promising alternative technology for the food processing industry.

## 1. Introduction

*Listeria monocytogenes* (*L. monocytogenes*) is a major foodborne pathogen capable of growth across 0–45 °C [1], causing listeriosis with manifestations including bacteremia, central nervous system infections, and perinatal complications [2], with mortality rates of 20–30% among immunocompromised individuals [3]. It is widely present in foods and environment, and prepared salads represent a high-risk food category for *L. monocytogenes* contamination and foodborne transmission [4].

Prepared salads typically lack a thermal processing step, creating a conducive environment for the proliferation of *L. monocytogenes* [5]. Among the pathogens contaminating these products, *L. monocytogenes* is a predominant concern [6]. Studies demonstrate its ability to maintain high populations in celery and chicken salads even during prolonged refrigeration [7].

Bacteria in the viable but non-culturable (VBNC) state cannot grow on conventional media, yet they remain alive [8]. The persistence of VBNC bacteria in food-processing environments poses a serious food safety concern, as these cells can evade routine detection methods while retaining pathogenic potential. Notably, VBNC *L. monocytogenes* still exhibit certain virulence, maintain stable toxin-gene expression, and can produce enterotoxins [9]. The VBNC state is particularly relevant in the context of sanitizer-induced stress, as sub-lethal concentrations of chemical disinfectants are well-documented triggers for VBNC formation in *L. monocytogenes* [10]. Systematic monitoring of the VBNC state is essential to comprehensively assess true bactericidal efficacy.

To control *L. monocytogenes* in prepared salads, traditional sanitization methods have been widely employed. However, they present significant limitations. For instance, some European countries prohibit chlorine usage in prepared foods, while alternative agents such as hydrogen peroxide may accelerate enzymatic browning in salad matrices [9]. Consequently, there is an urgent need to explore green, high-efficacy alternative sanitization technologies. Ultrasound (US) is a mechanical vibration wave enabling rapid sanitization with minimal food damage [11], though thermal effects limit its prolonged use [12]. Slightly acidic electrolyzed water (SAEW), primarily composed of hypochlorous acid [13], is recognized for its high antimicrobial efficiency, safety, and environmental friendliness [14]. Phenyllactic acid (PLA), a safe small-molecule phenylpropionic acid, serves as a novel broad-spectrum antibacterial biological preservative [15]. The combination of multiple sanitization techniques enables the development of more efficient pathogen control strategies. The cavitation effect of US treatment enhances the antimicrobial efficacy of other sanitization methods [16]. Liu et al. demonstrated that the combined application of SAEW and PLA sanitization reduced total plate count by approximately 4.5 log CFU/mL, as the two agents synergistically generated a slightly acidic environment that strengthened the bactericidal effect [17]. However, current studies have not clarified the combined bactericidal effect of ultrasound integrated with SAEW and PLA against *L. monocytogenes*. Furthermore, few studies have systematically evaluated the potential risk of VBNC state induction by this combined treatment in ready-to-eat salad matrices [8,10].

The rationale for combining US, SAEW, and PLA is grounded in their complementary mechanisms of action. SAEW exerts its bactericidal effect primarily through hypochlorous acid (HClO), which oxidizes bacterial cell membrane components and disrupts metabolic enzymes [13]. PLA destabilizes the phospholipid bilayer through hydrophobic interactions of its phenyl ring structure, increasing membrane permeability and facilitating the influx of antimicrobial agents [18]. US generates acoustic cavitation-induced micro-jets that physically disrupt cell membranes and enhance mass transfer of SAEW and PLA into bacterial cells [19]. Notably, both SAEW and PLA generate mildly acidic microenvironments (pKa of HClO ≈ 7.5) that favor the protonated, membrane-permeable form of HClO, thereby amplifying oxidative bactericidal activity [17]. This mechanistic complementarity provides a robust scientific basis for the proposed triple combination.

Based on the complementary modes of action of US, SAEW, and PLA, we hypothesize that their combination exerts a multi-target synergistic effect—integrating acoustic mechanical stress with severe chemical membrane permeabilization—to achieve superior bactericidal efficacy at reduced dosages. Furthermore, we hypothesize that this intense, simultaneous attack can irreversibly compromise cellular integrity, directly leading to rapid cell death and effectively preventing the bacteria from entering the VBNC survival state. To test these hypotheses, this study systematically evaluated the synergistic interactions on *L. monocytogenes*, elucidated the underlying antibacterial mechanisms through membrane-targeted physiological indicators and microscopic observations, and validated the practical efficacy of this strategy in maintaining the quality of prepared salads.

## 2. Materials and Methods

### 2.1. In Vitro Antimicrobial Experiments in Bacterial Suspension Systems

#### 2.1.1. Strain, Cultural Conditions and Sanitizer Preparation

Brain Heart Infusion (BHI) medium and Plate Count Agar (PCA) medium were purchased from Huankai Co., Ltd. (Guangzhou, China). *L. monocytogenes* ATCC 19115 was purchased from Guangdong Microbial Culture Collection Center (Guangzhou, China) and incubated in BHI medium at 37 °C (150 rpm) for 24 h. The total count was measured on PCA medium. A 10 mg/mL PLA solution was obtained by dissolving 10 g PLA powder (P102441; Aladdin Technology Co., Ltd., Shanghai, China) in 1000 mL distilled water. SAEW was generated by electrolyzing a 0.1% (v/v) HCl solution using a SAEW generator (Hanston Technology Co., Ltd., Changsha, China) with a working current of 2–8 A. The available chlorine concentration (ACC) of the freshly generated SAEW was determined by iodometric titration according to the Chinese national standard GB/T 26367-2010 [20]. The pH and oxidation-reduction potential (ORP) of SAEW were measured using a calibrated pH/ORP meter (Seven Compact S220; Mettler-Toledo, Columbus, OH, USA) immediately prior to each experiment. The SAEW generator can produce electrolyzed water with an available chlorine concentration (ACC) ranging from 10 to 80 mg/L. For all formal experiments in this study, SAEW working solutions with gradient ACC levels of 2.5, 5, 10, 20, and 40 mg/L were prepared by dilution. These working solutions maintained a stable pH range of 5.0–6.5 and an ORP value above 900 mV.

#### 2.1.2. Minimum Inhibitory Concentration (MIC) and Fractional Inhibitory Concentration Index (FICI) of SAEW and PLA

MIC was determined following Mattila et al. (2011) with modifications [21]. PLA (10 mg/mL) and SAEW (40 mg/L) were serially diluted using a two-fold dilution method. NaClO (40 mg/L) served as a control with an identical dilution procedure. Each dilution series included positive (bacteria only) and negative (medium only) controls. Treated bacterial suspensions were incubated at 37 °C for 24 h, and the optical density at 600 nm (OD_600_) was measured using a Bioscreen C automatic growth curve analyzer (Labsystems, Helsinki, Finland). FICI was determined by the checkerboard assay. SAEW and PLA were two-fold diluted to concentrations ranging from 1/8 to 4 MIC, plus 0 MIC. In 96-well plates, 100 μL the SAEW-PLA mixture and 100 μL of the bacterial suspension were mixed and incubated at 37 °C for 24 h. The OD_600_ was recorded by the Bioscreen C analyzer and the FICI was calculated according to Pollini et al. (2018) [22].

(1) $$\mathrm{FICI}={\mathrm{FIC}}_{\mathrm{SAEW}}+{\mathrm{FIC}}_{\mathrm{PLA}}=\frac{c_{\mathrm{SAEW}}^{\mathrm{comb}}}{{\mathrm{MIC}}_{\mathrm{SAEW}}^{\mathrm{alone}}}+\frac{c_{\mathrm{PLA}}^{\mathrm{comb}}}{{\mathrm{MIC}}_{\mathrm{PLA}}^{\mathrm{alone}}}$$

${\mathrm{MIC}\;}_{\mathrm{SAEW}}^{\mathrm{alone}}$ and ${\mathrm{MIC}\;}_{\mathrm{PLA}}^{\mathrm{alone}}$ are the MICs of single component SAEW and PLA respectively. ${c\;}_{\mathrm{SAEW}}^{\mathrm{comb}}$ and ${c\;}_{\mathrm{PLA}}^{\mathrm{comb}}$ are the effective concentrations of SAEW and PLA in the combination, respectively. The fractional inhibitory concentration index (FICI) was interpreted as follows: FICI ≤ 0.5 indicated synergy, 0.5 < FICI ≤ 4 indicated no interaction, and FICI > 4 indicated antagonism.

#### 2.1.3. Bactericidal Effects of SAEW and PLA on *L. monocytogenes*

*L. monocytogenes* suspensions at different initial concentrations (10^7^, 10^6^, and 10^5^ CFU/mL) were exposed to SAEW (40, 20, 10, 5, and 2.5 mg/L) or PLA (10, 5, 2.5, 1.25, and 0.625 mg/mL) for 5 min, with 0.85% saline used as the control. After treatment, the suspensions were centrifuged (8000 rpm, 5 min, 4 °C), and washed three times with sterile phosphate-buffered saline (PBS) (P1020, Solarbio Co., Ltd., Beijing, China), and the viable bacteria were quantified by standard plate counting [23].

#### 2.1.4. Bactericidal Effect of Ultrasonic Treatment on *L. monocytogenes*

This experiment was conducted as a screening to determine the optimal ultrasonic parameters for subsequent combined sanitization. The ultrasonic parameter optimization was conducted in two sequential steps: power gradient screening was first performed at a fixed treatment time of 5 min, and the selected power was then used for the subsequent time gradient evaluation. Different concentrations of *L. monocytogenes* (10^7^, 10^6^, and 10^5^ CFU/mL) were subjected to ultrasonic treatments at varying power levels of 0, 100, 200, 300, 400, 500, and 600 W (ultrasound frequency 40 kHz, SCIENTZ-IID, Xingzhi Co., Ltd., Ningbo, China) for a fixed duration of 5 min. Meanwhile, ultrasonic treatments at a constant power of 400 W were applied with varying times of 0, 5, 15, 20, 25, and 30 min [24]. The ultrasonic probe (diameter: 6 mm) was immersed to a depth of 1 cm below the liquid surface. All samples were placed on ice during US treatment to minimize thermal effects. Consequently, the temperature increase did not exceed 4 °C above the initial temperature as monitored by a thermocouple. Subsequently, the treated bacterial suspensions were centrifuged (8000 rpm, 5 min, 4 °C), and the cells were then washed three times with sterile PBS prior to standard plate counting [24].

#### 2.1.5. Bactericidal Effect of Combined Sanitization Method on *L. monocytogenes*

Combinations of different concentrations of SAEW, PLA, and 400 W US treatment were employed to treat *L. monocytogenes*. Specifically, seven paired formulations of SAEW and PLA solutions were prepared to evaluate their synergistic bactericidal effects. These combinations consisted of 1/4, 1/2, and 1 MIC SAEW blended with 1 MIC PLA, as well as 1/4, 1/2, 1, and 2 MIC SAEW mixed with 1/2 MIC PLA (Table 1). All solutions were filter-sterilized using 0.22 μm polyethersulfone syringe membrane filters (Millipore, Burlington, MA, USA) before use.

Then the mixture and US (400 W) were used simultaneously to treat *L. monocytogenes* for 5 min. Subsequently, the treated bacterial suspensions were promptly centrifuged (8000 rpm, 5 min, 4 °C), and the cells were washed three times with sterile PBS. Viable *L. monocytogenes* cells were enumerated as described previously [25].

#### 2.1.6. Determination of VBNC State of *L. monocytogenes* During Sanitization Process

Four experimental groups (SAEW alone, PLA alone, SAEW/PLA combination, and US/SAEW/PLA combined sanitization) were designed based on the previously determined MICs. Three concentration levels of 1/4 MIC, 1/2 MIC, and 1 MIC were selected to cover a range from sub-lethal to lethal exposure, as sub-lethal treatment is the primary trigger for VBNC state formation. This stepwise design clarifies the VBNC induction patterns of the individual agents and verifies whether the multi-hurdle sanitization strategy can effectively eliminate VBNC risks.

A 20 mL *L. monocytogenes* suspension (10^7^ CFU/mL) was centrifuged (8000 rpm, 5 min, 4 °C) and washed three times with sterile PBS. The harvested cells were then subjected to the aforementioned four treatment series. For all chemical treatments, the sanitizer contact time was fixed at 5 min. For the combined sanitization groups, the chemical exposure was coupled with simultaneous ultrasound treatment at a power of 400 W for 5 min. Then, the cells were collected by centrifugation (8000 rpm, 5 min, 4 °C) and washed again as described above. Culturable and total viable bacterial populations were quantified using standard plate counting and PMA-qPCR [26]. The development of the PMA-qPCR assay is detailed in Figures S1–S3 and Tables S1 and S2.

#### 2.1.7. Effects of Combined Sanitization Method on ANS Fluorescence, Zeta Potential, ONPG Permeability and Extracellular Protein Content

A 20 mL *L. monocytogenes* suspension (10^7^ CFU/mL) was centrifuged (6000 rpm, 10 min, 4 °C) and washed three times with sterile PBS. A 10 mL aliquot was treated with the combined or individual methods for 5 min. Subsequently, the treated suspensions were centrifuged (8000 rpm, 5 min, 4 °C) and resuspended in sterile PBS for the following assays:

For membrane fluidity, the suspension was mixed with 20 μL of 8-aniline-1-naphthalenesulfonic acid (ANS) (8 mmol/L) and incubated in the dark for 30 min. Fluorescence was measured using a spectrophotometer (SpectraMax i3x; Molecular Devices, Sunnyvale, CA, USA) at excitation and emission wavelengths of 385 nm and 473 nm, respectively [27].

Zeta potential was measured using a Zetasizer Nano ZS 90 (Malvern Panalytical, Malvern, UK) [28].

To evaluate membrane permeability, a 30 μL aliquot of the treated suspension was mixed with 600 μL of ortho-nitrophenyl-β-D-galactopyranoside (ONPG) solution (30 mmol/L) and incubated in the dark for 30 min, after which OD_420_ was measured using a microplate reader. Extracellular protein content was determined according to the manufacturer’s instructions using a Bradford Protein Assay Kit (PC0010; Solarbio Co., Ltd., Beijing, China) [29].

#### 2.1.8. Observation of *L. monocytogenes* by Inverted Fluorescence Microscopy and Scanning Electron Microscopy (SEM)

After *L. monocytogenes* was treated with the combined sanitization method (as described in Section 2.1.5), the LIVE/DEAD bacterial staining kit (FS4005; Fusheng Co., Ltd., Shanghai, China) was used to stain the cells according to the manufacturer’s instructions. A 10 μL of the stained bacterial suspension was observed under an inverted fluorescence microscope (Axio Observer A1; Zeiss, Oberkochen, Baden-Wurttemberg, Germany) [30].

For SEM observation, the cells were fixed in 2.5% phosphate-buffered glutaraldehyde for 24 h, then dehydrated through a graded ethanol series and subjected to critical point drying. After gold sputter-coating for 30 s, the samples were examined with a scanning electron microscope (SU8020; HITACHI, Tokyo, Japan). The accelerating voltage was set at 5 kV, and the SEM point resolution was 50 nm [31].

#### 2.1.9. Detection of *L. monocytogenes* by Flow Cytometry

According to the instructions for the LIVE/DEAD bacterial staining kit, 1.5 L SYTO 9 and 1.5 μL PI were added to 1 mL of a bacterial suspension that was treated with the combined sanitization method (refers to 2.5). After incubation in the dark for 15 min, cell viability was measured using flow cytometry (Cytoflex; Beckman Coulter, Miami, FL, USA) [32].

### 2.2. In Situ Experiments in Prepared Salad

#### 2.2.1. Preparation of Prepared Salads

Fresh ingredients were purchased from a local supermarket (Guangzhou, China) and stored at 4 °C for 12 h before experimental use to ensure maximum physiological consistency. Cooked chicken breast (steamed at 100 °C for 20 min to achieve an internal temperature ≥ 75 °C) was used in this study. The prepared salad consisted of 90 g head lettuce, 15 g Rosa lettuce, 15 g Spanish green lettuce, 5 g shredded purple cabbage, 8 g shredded carrots, 8 g cucumber slices, 10 g radish slices, 5 g corn kernels, 23 g cherry tomatoes, and 20 g cooked chicken breast. The salad components were carefully selected based on their original preparation proportions to maintain compositional consistency throughout the study. All salad samples were uniformly cut into 2 × 1 cm pieces using autoclaved tools to ensure consistent treatment exposure. Prior to inoculation or sanitization treatment, all vegetable pieces were gently rinsed with sterile distilled water to remove surface dust and broken cell debris, then blotted dry with sterile filter paper to remove excess surface water and ensure uniform initial moisture content across all samples. Following different sanitization treatments, the samples were drained, placed into sterile polyethylene zip-lock bags (200 mL capacity), sealed under an ambient condition, and stored at either 4 °C or 10 °C for a maximum of 9 days. Sampling was conducted every 3 days over a 9-day storage period to measure the parameters described below.

#### 2.2.2. Total Plate Count and the Amount of *L. monocytogenes*

To reduce the native background microbiota prior to artificial bacterial inoculation, a preliminary UV-C decontamination step was applied. Salad components in a sterile stainless steel tray were irradiated for 5 min using two 30 W UV-C lamps (254 nm; G30T8, Philips, Amsterdam, The Netherlands). Agitation was maintained at 120 rpm using an orbital shaker (SK-O330-Pro, DLAB Scientific, Beijing, China) at a fixed lamp-to-sample distance of 20 cm. For the CK group, the inoculated salad samples were treated with sterile 0.85% physiological saline under identical conditions, instead of the experimental sanitizers.

##### Determination of Total Plate Count

A 25 g salad sample was mixed with 225 mL of sterile PBS and homogenized (FSH-2A; Jingfei Co., Ltd., Beijing, China) at 18,000 rpm for 2 min. The homogenate was inoculated onto PCA medium and incubated at 36 °C ± 1 °C for 48 h to determine the total plate count.

##### Determination of *L. monocytogenes* in Inoculated Samples

A 25 g salad sample was soaked in an *L. monocytogenes* suspension for 3 min to achieve an initial inoculum of 10^2^–10^3^ CFU/g. Following the combined sanitization treatment, samples were homogenized for 2 min and inoculated onto PALCAM agar. Plates were incubated at 36 °C ± 1 °C for 48 h to enumerate the *L. monocytogenes* count [33].

#### 2.2.3. Changes in Moisture Content and Weight Loss Rate

A 10 g salad sample was dried at 105 °C for 4 h to constant weight, and the resulting weight change was recorded as moisture content. The weight loss ratio was determined every 3 days according to previously reported methods [34].

#### 2.2.4. Changes in Soluble Solids Content, Texture and Color of Prepared Salads

After homogenization, the salad juice was filtered through four layers of standard medical degreased gauze (20 × 20 cm) to remove large tissue fragments and insoluble fiber particles prior to soluble solids measurement. The nominal equivalent pore size of four-layer stacked gauze is approximately 18 μm, which is a widely accepted specification for this standard pretreatment in quality analysis of fresh-cut produce [35]. This filtration procedure only removes macroparticles and does not interfere with the quantification of soluble solids. The soluble solids content was measured using a handheld refractometer (LB32T; Minrui Co., Ltd., Guangzhou, China) [36].

The texture was evaluated using a texture analyzer (TA.XTPlus; SMS, Bletchingley, Surrey, UK) in texture profile analysis (TPA) mode with a shear probe (P/5), at a test speed of 100 mm/min, a 50% deformation rate, and a 40 mm return distance. Each sample was measured at three equidistant points, and shear force ratios were used to indicate texture changes [37].

Color measurement was performed on the surface of lettuce leaves to avoid compositional heterogeneity of the mixed salad samples. Lettuce was selected as the representative matrix owing to its highest proportion in the salad formula and its high susceptibility to enzymatic browning, which is a primary factor driving consumer sensory rejection.

A colorimeter (ST70; 3nh Co., Ltd., Shenzhen, China) equipped with an 8 mm diameter measuring aperture was used for color determination. The instrument was calibrated with a standard white tile and a zero-calibration black cup before each batch of measurements to ensure data accuracy. For each sample, three flat and intact leaf surfaces were randomly selected (avoiding main veins), and each site was measured once. The average of the three readings was calculated to represent the color attribute of the sample, with results expressed as L* (lightness), a* (redness/greenness), and b* (yellowness/blueness) values [38]. The total color difference (ΔE) was calculated as follows:

$$\Delta E=\sqrt{(\Delta L^{*}{)}^{2}+(\Delta a^{*}{)}^{2}+(\Delta b^{*}{)}^{2}}$$

#### 2.2.5. Electronic Nose Measurement

A 10 g salad sample in a 50 mL headspace vial was analyzed using an electronic nose (PEN3; AIRSENSE, Schwerin, Germany). Data were evaluated using principal component analysis (PCA) and radar plots [39].

#### 2.2.6. Determination of Catalase (CAT) and Peroxidase (POD) Activities

CAT and POD activities were assayed using corresponding commercial kits (BC0205 and BC0095; Solarbio Co., Ltd., Beijing, China). For CAT, 10 μL sample extract was mixed with 190 μL kit working solution (substrate-buffer system) in a 96-well plate; OD_240_ was recorded initially and after 1 min. For POD, 5 μL sample extract was mixed with 240 μL kit test solution (phenolic substrate system), and 200 μL was transferred to a 96-well plate [24]. OD_470_ was recorded at 30 s and 90 s. Enzyme activities were calculated from absorbance changes [40].

### 2.3. Data Analysis

Data were analyzed and plotted using SPSS 25.0 (IBM, New York, NY, USA) and GraphPad Prism 8.0 (GraphPad Software, San Diego, CA, USA). Statistical significance was assessed using one-way analysis of variance (ANOVA) followed by Tukey’s post hoc test or Student’s *t*-test. Data are expressed as mean ± SD (*n* = 3), and different letters denote significant differences (*p* < 0.05).

## 3. Results and Discussion

### 3.1. In Vitro Antimicrobial Evaluation in Bacterial Suspension Systems

#### 3.1.1. MIC and FICI

MICs for SAEW and PLA were 10 mg/L and 2.5 mg/mL, respectively. The FICI (0.5) indicated synergism, validated by the significantly enhanced bactericidal efficacy of the combination compared to individual treatments (Table S3).

#### 3.1.2. Effects of SAEW and PLA on *L. monocytogenes* at Different Concentrations

**Figure 1:**
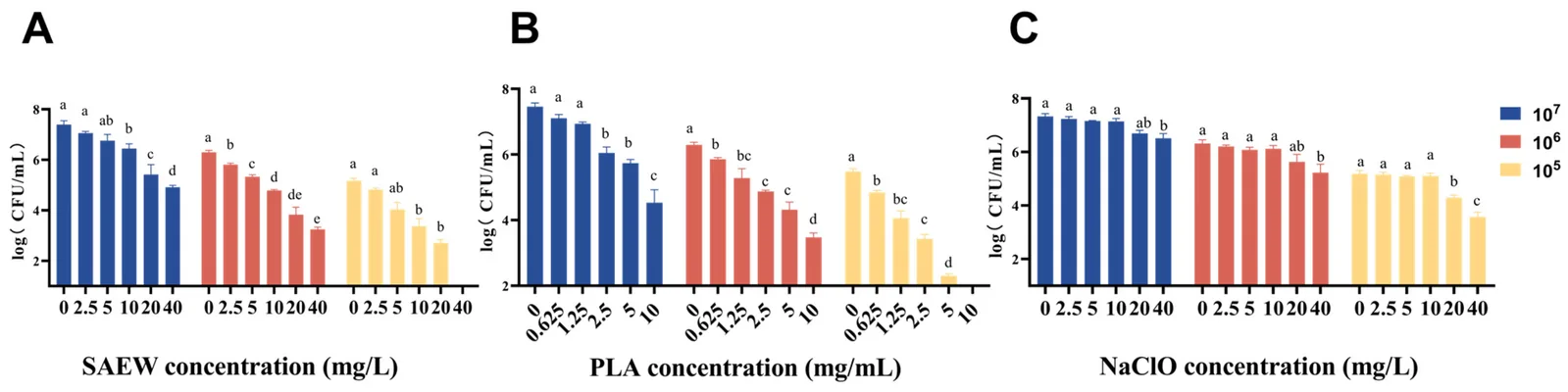
Bactericidal effects of different sanitizers on *L. monocytogenes* at various concentrations. (**A**) SAEW; (**B**) PLA; (**C**) NaClO. The treatment time was 5 min. Different lowercase letters indicate significant differences among treatments according to Tukey’s test (*p* < 0.05).

Bactericidal effects of SAEW and PLA decreased as initial bacterial concentrations increased. Following a 5 min treatment with 40 mg/L SAEW, viable counts for 10^7^ and 10^5^ CFU/mL suspensions decreased by 2.50 and 5.18 log CFU/mL, respectively (Figure 1A). Similarly, a 5 min treatment with 10 mg/mL PLA yielded reductions of 2.63 and 5.49 log CFU/mL, respectively (Figure 1B). Compared with NaClO (Figure 1C), SAEW showed superior bactericidal efficacy under identical conditions.

#### 3.1.3. Effect of US Treatment on *L. monocytogenes*

**Figure 2:**
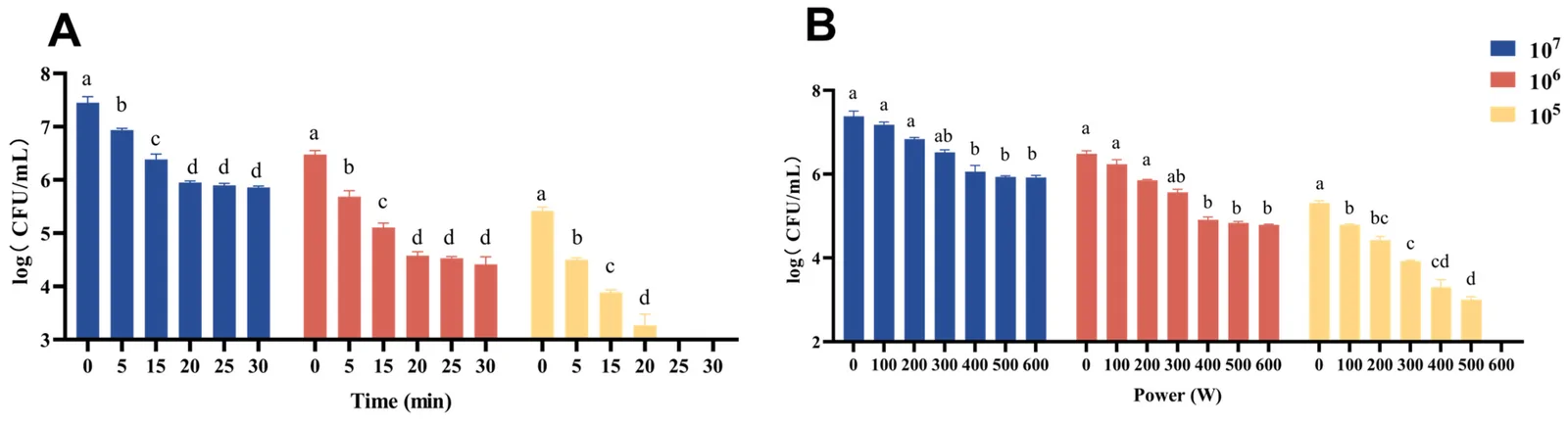
Bactericidal effect of ultrasound (US) treatment on *L. monocytogenes* at different initial concentrations (10^7^, 10^6^, and 10^5^ CFU/mL). (**A**) Effect of different treatment times at a fixed ultrasound power of 400 W. (**B**) Effect of different ultrasound powers at a fixed treatment time of 5 min. Different lowercase letters indicate significant differences among treatments within the same initial concentration group according to Tukey’s test (*p* < 0.05).

The bactericidal efficacy of US was inversely correlated with the initial concentration of *L. monocytogenes*. However, regardless of the initial concentration of 10^7^, 10^6^, or 10^5^ CFU/mL, significant reductions in viable counts were achieved after 5 min of US treatment at a fixed power of 400 W. Further significant reductions were observed when the treatment time was prolonged to 15 and 20 min, showing a distinct time-dependent bactericidal effect (Figure 2). These results are consistent with previous studies showing that higher ultrasonic power and longer treatment improve the inactivation of *L. monocytogenes* via enhanced acoustic cavitation and cell membrane damage [41]. The lower disinfection efficiency observed at higher initial bacterial loads can be explained by cell aggregation, which shields inner cells from cavitation stress. Accordingly, 400 W and 5 min were chosen as the optimal conditions to strike a balance between bactericidal efficacy, energy consumption, and thermal-related quality risk.

#### 3.1.4. Effects of Combined Sanitization Method on *L. monocytogenes*

**Figure 3:**
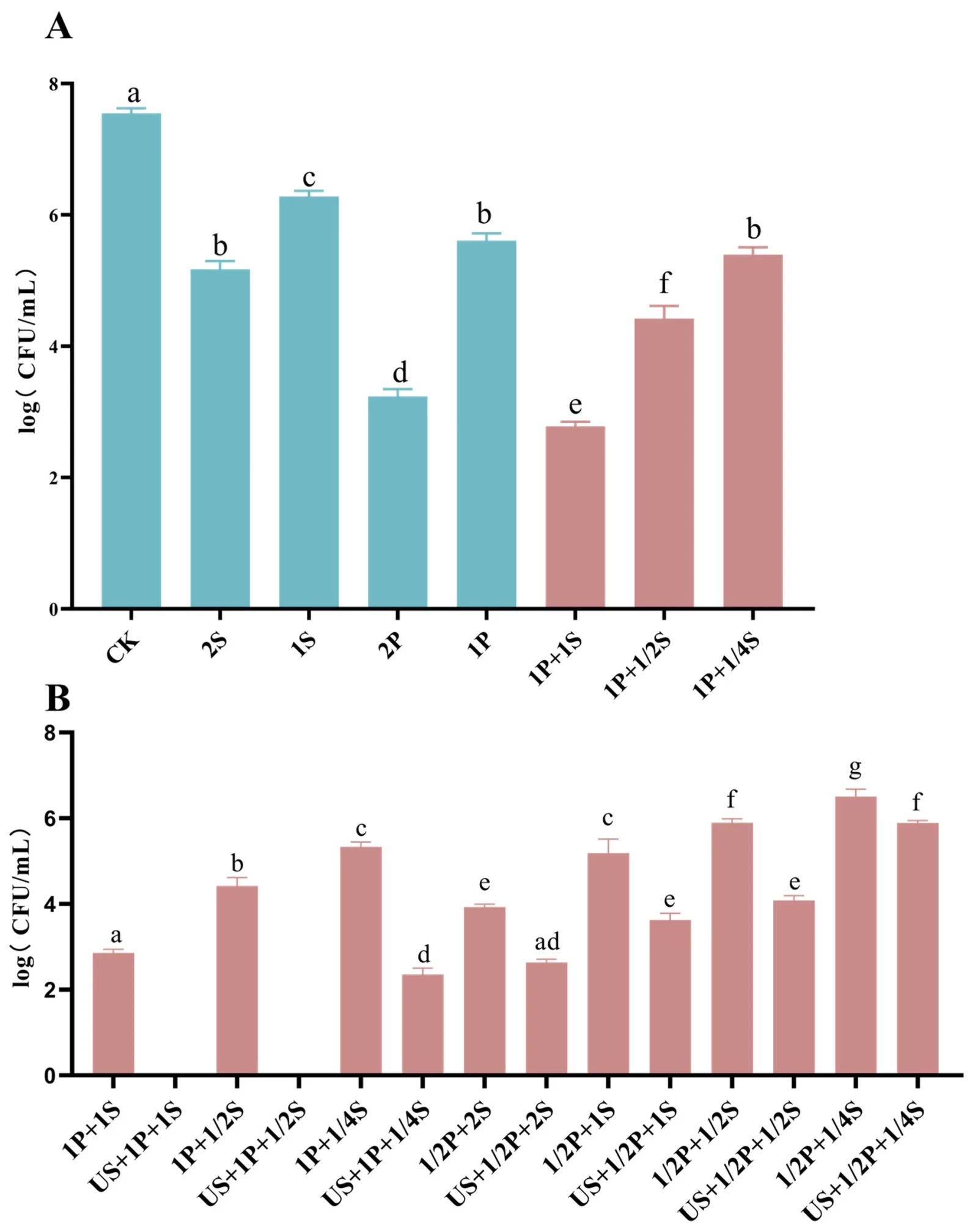
Effects of combined sanitization methods on *L. monocytogenes*. (**A**) Synergistic effect of SAEW and PLA. (**B**) Combined effect of SAEW, PLA, and US. ND indicates ‘Not Detected’ (bacterial counts were below the detection limit). Different lowercase letters indicate significant differences among treatments according to Tukey’s test (*p* < 0.05).

Combined treatments exhibited potent bactericidal effects on *L. monocytogenes*. Viable counts in 1P + 1S group were 2.78 log CFU/mL, significantly lower than the 3.24 log CFU/mL in 2S group. This enhanced efficacy of the dual combination aligns with the synergistic FICI results (Figure 3A). US integration significantly reduced viable counts compared to non-US treatments. For example, counts in the US + 1/2P + 1S group were 3.63 log CFU/mL, significantly lower than 5.45 log CFU/mL in the 1/2P + 1S group. Similarly, counts in the US + 1P + 1/2S group were significantly lower than 5.99 log CFU/mL in the 1P + 1/2S group (Figure 3B). This synergy is presumably attributed to the mildly acidic environment generated by SAEW and PLA, which increases the susceptibility of bacterial cells to oxidative stress [17,19,41]. Furthermore, the integration of US creates high-energy cavitation micro-environments, which act as a physical driver to facilitate the penetration and action of SAEW and PLA into the bacterial cells, thereby maximizing the sanitization efficiency [41]. For example (at 10^7^ CFU/mL), the reductions from 1/2P + 1S (1.55 log) and US alone (1.0 log) yield a theoretical additive reduction of 2.55 log CFU/mL. However, the US + 1/2P + 1S treatment achieved an actual reduction of 3.37 log CFU/mL. This significant enhancement confirms that acoustic cavitation effectively potentiates the chemical agents, achieving multi-target synergistic inactivation.

#### 3.1.5. VBNC State of *L. monocytogenes* Under the Combined Sanitization Treatments

**Figure 4:**
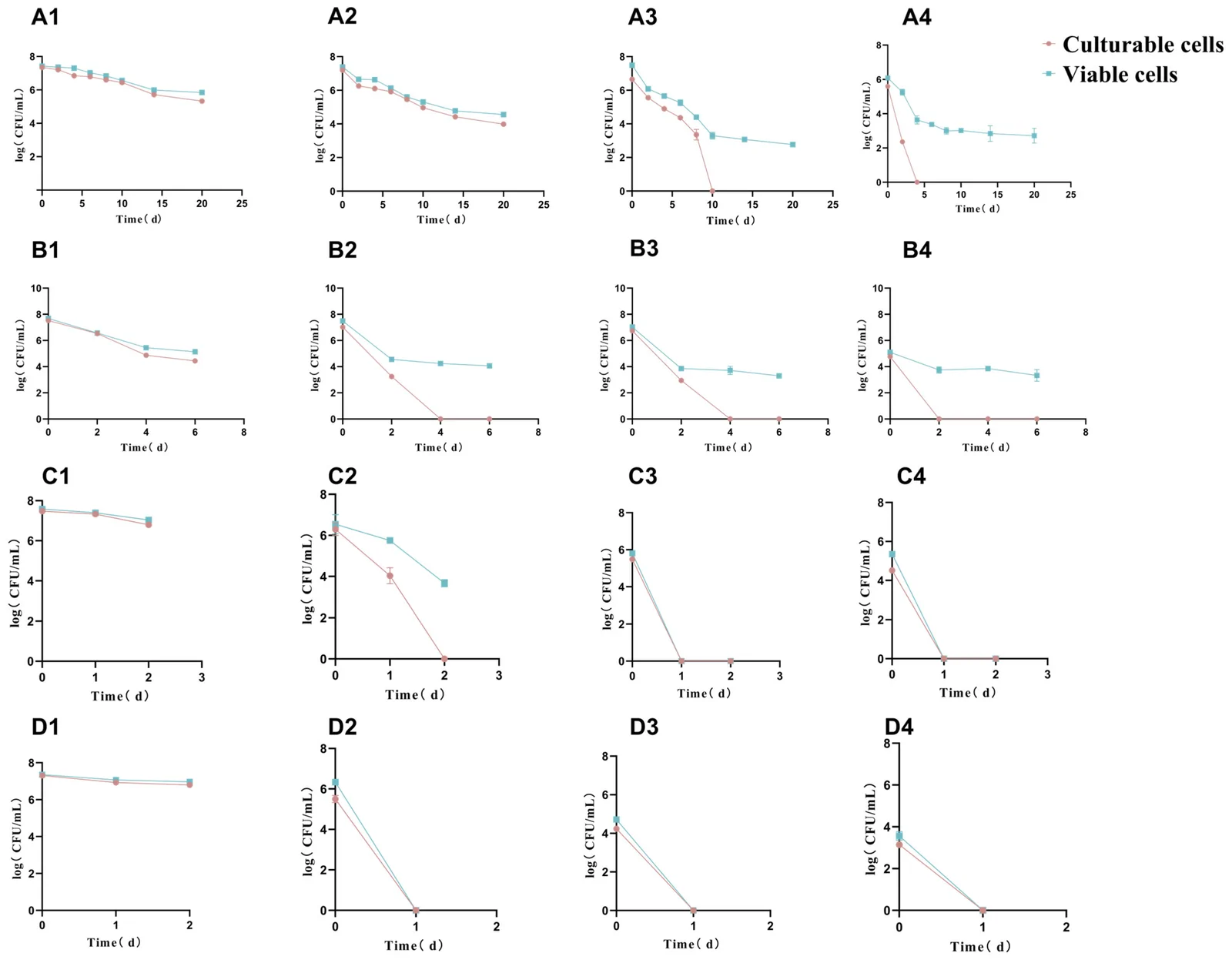
VBNC state of *L. monocytogenes* under different treatments. (**A1**–**A4**) SAEW alone (0, 1/4, 1/2, and 1 MIC); (**B1**–**B4**) PLA alone (0, 1/4, 1/2, and 1 MIC); (**C1**–**C4**) 1/2 MIC PLA combined with 0, 1/4, 1/2, and 1 MIC SAEW; (**D1**–**D4**) US + 1/2 MIC PLA combined with 0, 1/4, 1/2, and 1 MIC SAEW. The x-axis represents the post-treatment observation time (days). Data are presented as the mean ± standard deviation (SD) of three independent replicates. Error bars indicate SD, and statistical significance was set at *p* < 0.05.

Treatment with 1/2S induced the VBNC state in all cells within 10 days (3.30 log CFU/mL) (Figure 4(A3)). Conversely, 1/4P and 1/2P induced the VBNC state within 4 days, yielding VBNC cell counts of 4.12 and 3.38 log CFU/mL, respectively (Figure 4(B2, B3)). The 1/2P + 1/4S binary treatment accelerated VBNC transition to 2 days (3.67 log CFU/mL) (Figure 4(C2)). Notably, the triple combination (US + PLA + SAEW) completely suppressed VBNC induction, with no VBNC cells detected throughout the experimental period (Figure 4(D2–D4)).

Traditional food sanitizers often induce the VBNC state in pathogens, posing a critical food safety risk because these non-culturable cells evade detection by standard plate counting while retaining pathogenic potential [42]. Our results showed that binary combinations, especially SAEW and PLA, accelerated VBNC formation compared with single treatments. This effect may be attributed to the additive action of two sub-lethal stresses: mild oxidative damage caused by SAEW and hydrophobic membrane disruption induced by PLA. Together, these stresses activate the bacterial general stress response, and trigger energy-saving survival mechanisms without causing lethal cellular damage, thereby promoting VBNC formation [9,10]. In contrast, the triple treatment completely prevented VBNC transition by inducing direct bacterial death. The simultaneous application of ultrasonic physical rupture, chemical oxidation, and membrane destabilization caused irreversible cellular damage before bacteria could activate stress-defense pathways. This multi-hurdle strategy therefore effectively eliminates the false-negative risk associated with traditional chlorine-based sanitization, which may leave VBNC cells with retained pathogenicity.

#### 3.1.6. Effects of Combined Sanitization Method on ANS Fluorescence, Zeta Potential, ONPG Permeability and Extracellular Protein Content

**Figure 5:**
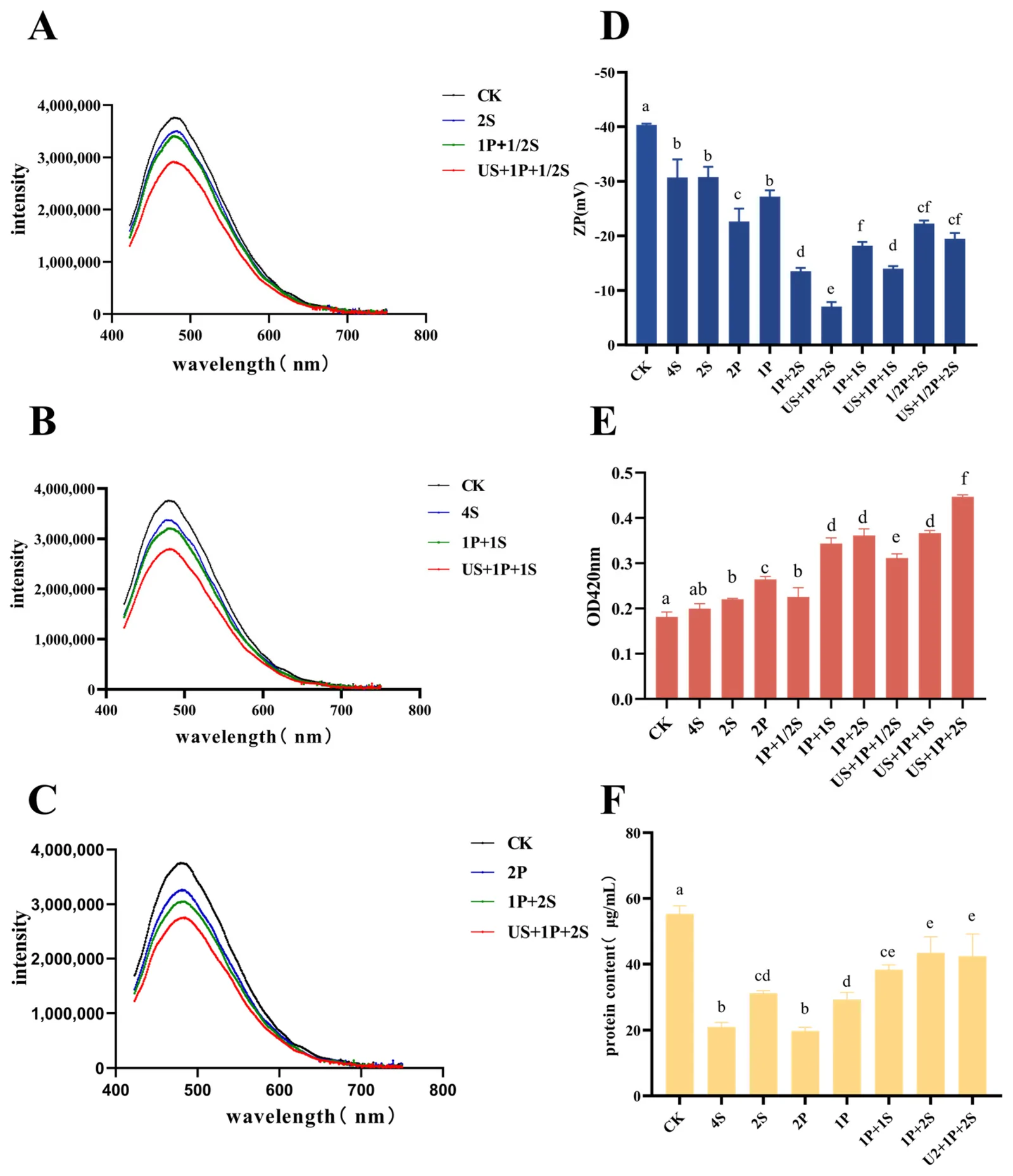
Changes in cellular characteristics of *L. monocytogenes* after different sanitization treatments. (**A**–**C**) ANS fluorescence intensity; (**D**) Zeta potential; (**E**) ONPG permeability (OD_420nm_); (**F**) Extracellular protein content. Different lowercase letters indicate significant differences among treatments according to Tukey’s test (*p* < 0.05).

ANS fluorescence intensity decreases when increased membrane fluidity hinders its binding. Following combined treatments, fluorescence intensity decreased significantly (Figure 5A–C) [43].

Increased membrane permeability causes intracellular ion leakage, altering zeta potential. The zeta potential of the CK group was −40.5 mV, compared to −7.06, −14.0, and −19.5 mV in the US + 1P + 2S, US + 1P + 1S, and US + 1/2P + 2S groups, respectively (Figure 5D). As cell membrane permeability increased, ONPG efficiently penetrates the membrane, leading to the increase in UV intensity at 420 nm [44]. The OD_420nm_ of the CK group was 0.181, lower than 0.358 of the 1P + 1S group and 0.447 of the US + 1P + 1S group (Figure 5E). The CK group exhibited the highest extracellular protein concentration due to continuous protein secretion by intact viable cells. In the 4S group and other single-treatment groups, extracellular protein levels decreased significantly. This phenomenon was probably caused by reduced bacterial metabolic activity, halted protein secretion, and the loss of membrane-associated proteins. Conversely, the combined treatments caused severe membrane rupture and significant intracellular protein leakage [45]. Consequently, extracellular protein concentrations in these groups were markedly higher than those observed under single treatments (Figure 5F), yet still lower than the level derived from active secretion in the untreated control.

Collectively, these membrane-related biomarkers reveal a multi-target synergistic bactericidal pathway. Initially, US cavitation creates surface micro-pores via mechanical shear, effectively lowering penetration barriers [46]. Subsequently, SAEW-derived HClO disrupts the bacterial envelope by degrading cell wall peptidoglycan and membrane phospholipids [13,47]. This compromised barrier facilitates the rapid influx of PLA. Once inside, PLA fluidizes the membrane (reflected by decreased ANS fluorescence), disrupts intracellular pH homeostasis, and inhibits key metabolic enzymes by binding to their sulfhydryl groups [18]. Crucially, this membrane fluidization further accelerates HClO diffusion into the cytoplasm, where it thoroughly oxidizes functional proteins and nucleic acids [47]. Phenotypically, this cascade manifests as surface charge imbalance (reduced absolute zeta potential), hyperpermeability (elevated ONPG), and massive intracellular protein leakage. Ultimately, this sequential physical–chemical damage explains why the multi-hurdle strategy achieves far stronger bactericidal performance than the simple additive effect of individual treatments.

#### 3.1.7. Observation of *L. monocytogenes* by Inverted Fluorescence Microscopy and Scanning Electron Microscopy

**Figure 6:**
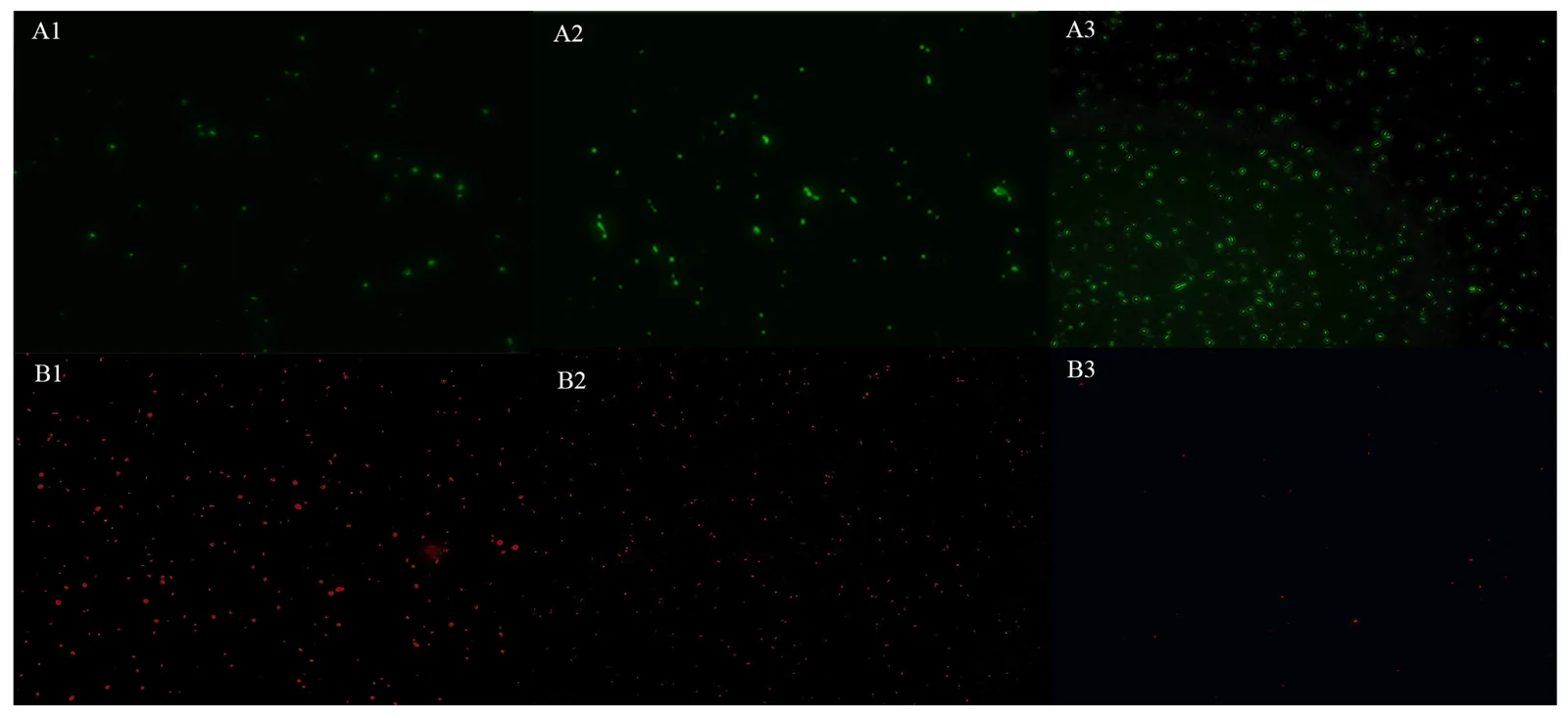
Inverted fluorescence microscopy images of *L. monocytogenes* (400×). (**A1**–**A3**) Live cells (green fluorescence). (**B1**–**B3**) Dead cells (red fluorescence). Treatments: (1) US + 1P + 1S; (2) 2S; (3) CK.

Fluorescence microscopy revealed predominantly dead cells following the US + 1P + 1S treatment. Conversely, *L. monocytogenes* treated with 1/2S exhibited more viable cells, indicating the presence of VBNC cells (Figure 6).

**Figure 7:**
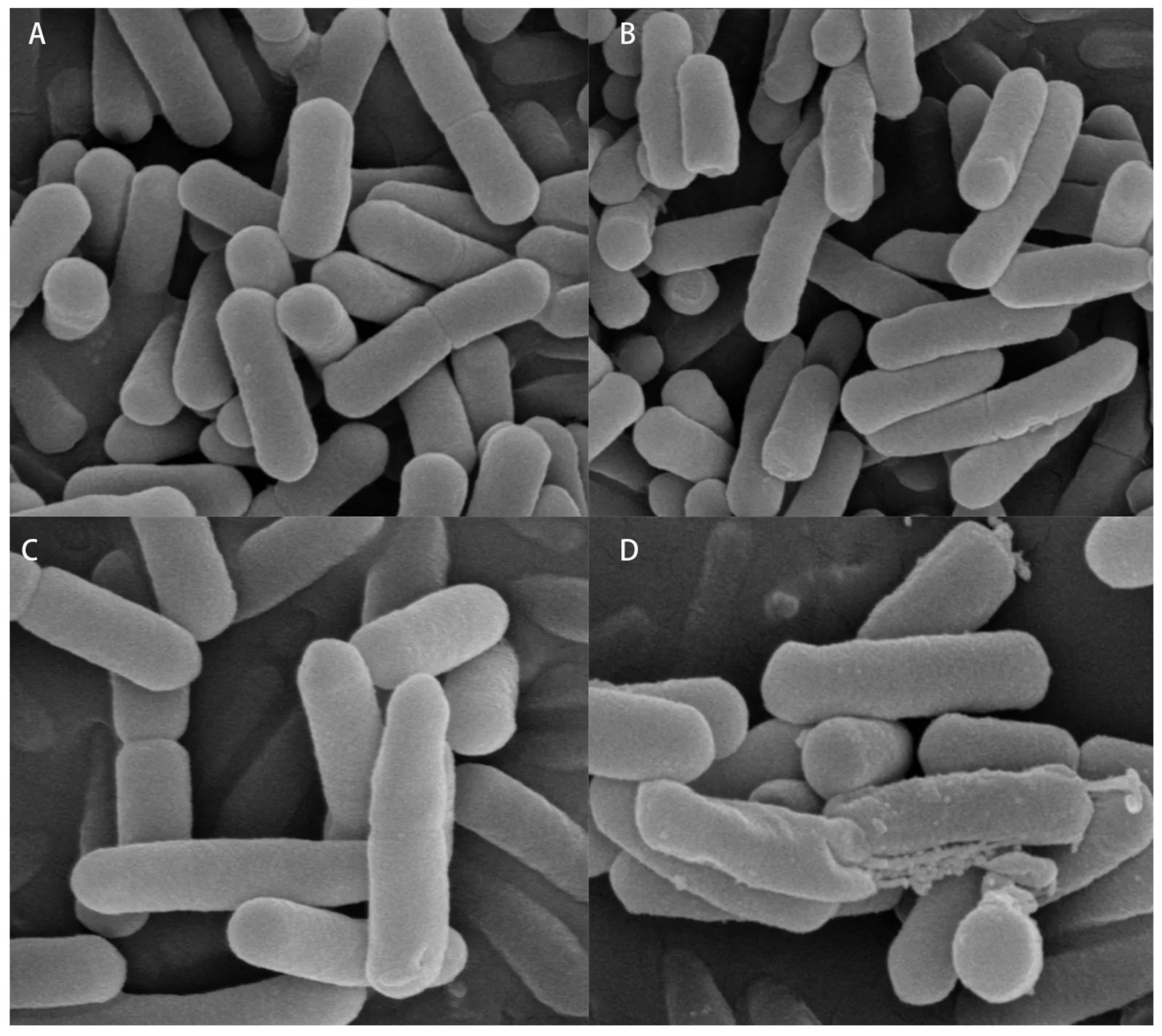
Scanning electron microscopy (SEM) images of *L. monocytogenes* (5000×). (**A**) CK; (**B**) 2S; (**C**) 1/2P + 1/2S; (**D**) US + 1/2P + 1/2S.

Scanning electron microscopy (SEM) showed that the control cells retained an intact, smooth, and regular short-rod morphology. In contrast, treatment with 2S caused surface wrinkling and depressions. The 1/2P + 1/2S binary treatment led to more pronounced surface deformation and loss of the rod-like shape. Ultimately, the US + 1/2P + 1/2S ternary treatment resulted in severe cell rupture, pore formation, and extensive damage to the cell envelope (Figure 7).

These microscopy analyses effectively visualize the transition from physiological impairment to terminal structural collapse [47]. The transition from green to red fluorescence and the observed loss of rod morphology corroborate the quantitative findings of reduced viability and increased membrane permeability, confirming that the combined sanitization method achieves its efficacy through irreversible physical and chemical disruption of the cell envelope.

#### 3.1.8. Detection of *L. monocytogenes* by Flow Cytometry

**Figure 8:**
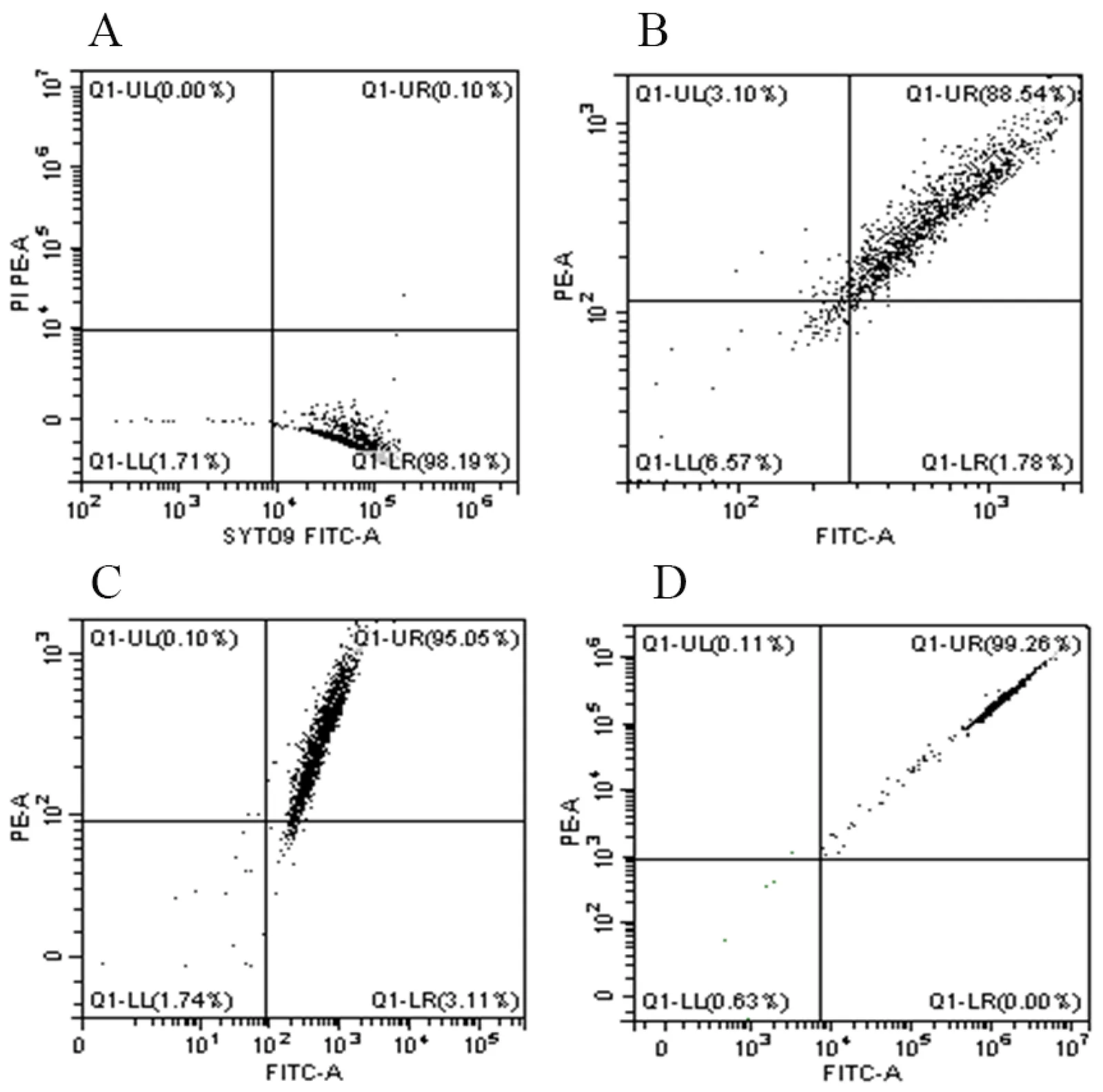
Flow cytometry analysis of *L. monocytogenes* viability after different sanitization treatments. (**A**) CK; (**B**) 2S; (**C**) 1/2P + 1/2S; (**D**) US + 1/2P + 1/2S.

The Q1-UR quadrant indicates dead cells stained by both SYTO-9 and PI, while the Q1-LR quadrant indicates viable cells stained only by SYTO-9. The mortality rate of *L. monocytogenes* in the US + 1/2P + 1/2S group reached 99.26%, significantly higher than that of the 2S single treatment (88.54%) or the 1/2P + 1/2S binary combination (95.05%) (Figure 8).

Collectively, the US + SAEW + PLA triple strategy exerts enhanced synergistic bactericidal efficacy against *L. monocytogenes*. SAEW and PLA show chemical synergism (FICI = 0.5), and US further provides physical potentiation via acoustic cavitation. Mechanistically, the treatment causes irreversible cell membrane damage and completely suppresses the induction of VBNC state. The US + 1P + 1S protocol was selected for subsequent validation experiments on salad samples based on three selection criteria: (1) highest bactericidal activity among all groups; (2) complete elimination of VBNC-related safety risk; (3) balanced reagent dosage to minimize adverse impacts on food quality.

### 3.2. In Situ Validation Results in Prepared Salad

#### 3.2.1. Total Plate Counts and *L. monocytogenes* Enumeration

The 4S group was set as a high-dosage positive control representing conventional high-dose chlorine-based sanitization, to benchmark the efficacy of the low-dose triple combination strategy. The 9-day refrigerated storage trial was designed according to the typical commercial shelf life of ready-to-eat fresh-cut salads [48]. Physicochemical and enzymatic tests were selected as core quality indicators, since moisture retention, texture firmness, color stability and antioxidant enzyme activity directly determine consumer acceptability and product shelf life.

**Figure 9:**
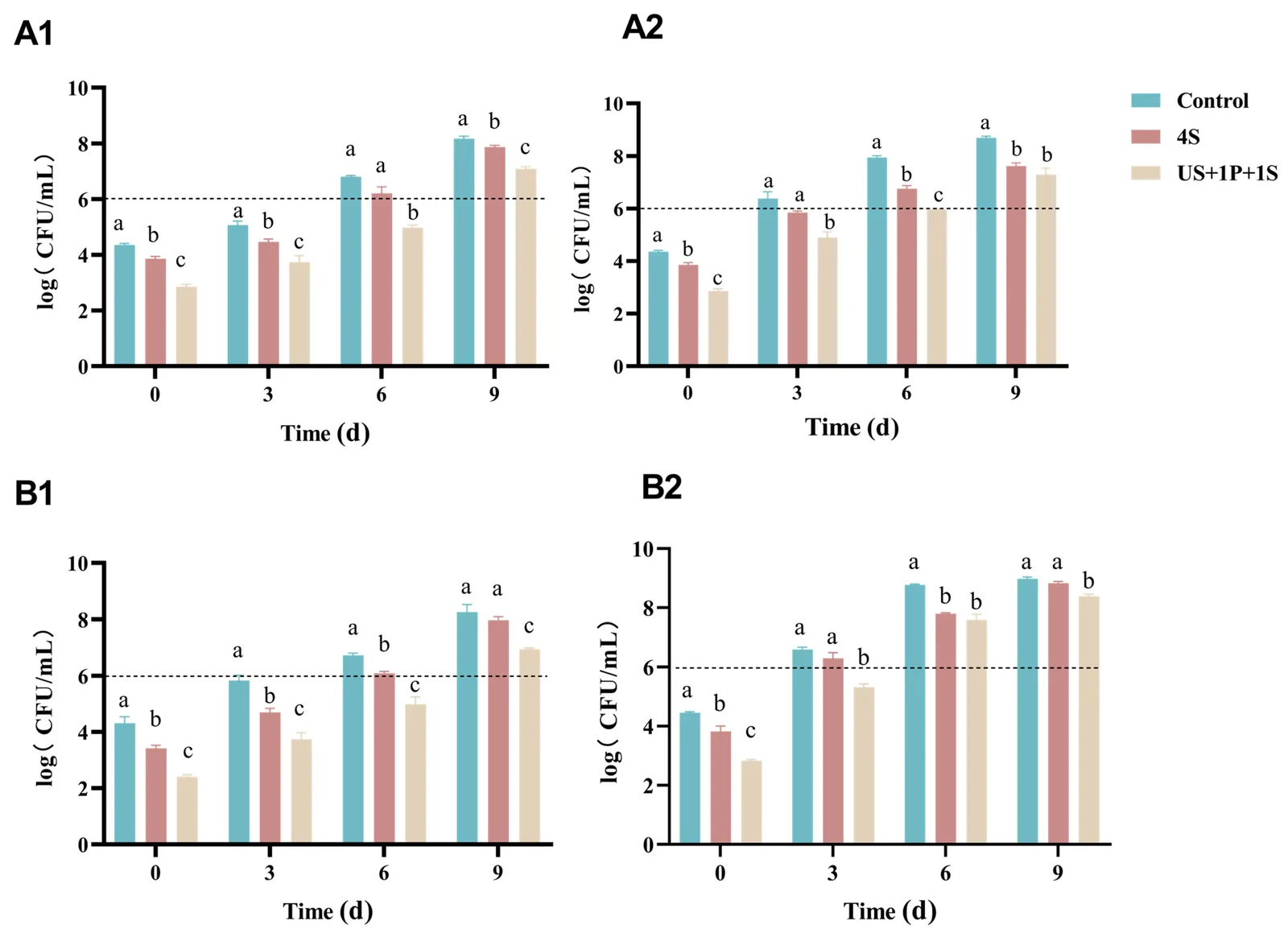
Changes in microbial populations in prepared salads during storage. (**A1**,**A2**) Total plate counts; (**B1**,**B2**) *L. monocytogenes* counts. Storage conditions: (1) 4 °C; (2) 10 °C. The horizontal dashed line indicates the maximum allowable limit (6 log CFU/g). Different lowercase letters indicate significant differences among treatments (*p* < 0.05).

The US + 1P + 1S treatment effectively reduced total plate counts in prepared salads, maintaining significantly lower bacterial populations than the CK and 4S groups at both 4 °C and 10 °C. The time required for total plate counts to exceed the maximum allowable limit (6 log CFU/g) depended on the storage temperature. During storage at 4 °C, both the CK and 4S groups exceeded this threshold on day 6, whereas the US + 1P + 1S group effectively delayed this until day 9. At 10 °C, the CK group spoiled more rapidly, exceeding the limit on day 3, followed by the 4S group on day 6. Remarkably, the US + 1P + 1S treatment successfully maintained the counts just below this critical threshold on day 6, only exceeding it on day 9 (Figure 9A).

Additionally, the US + 1P + 1S method effectively suppressed *L. monocytogenes* growth. During storage at 4 °C, *L. monocytogenes* counts in the US + 1P + 1S group were significantly lower than those in the CK and 4S groups from day 0 to day 9. Similarly, at 10 °C, counts in the US + 1P + 1S group remained significantly lower than the CK from days 0 to 9, and lower than the 4S group on days 0 and 3 (Figure 9B).

The bactericidal efficacy observed in the salad matrix was lower than that obtained in the in vitro suspension system, which can be attributed to several protective factors supporting bacterial survival on produce surfaces. Bacterial attachment to plant tissue and entrapment within surface micro-structures (stomata, cut edges) physically shield cells from sanitizer penetration, while surface organic matter partially neutralizes SAEW active chlorine and restricts antimicrobial diffusion [48]. From a food safety perspective, *L. monocytogenes* levels in the US + 1P + 1S group remained below the 100 CFU/g ready-to-eat limit throughout the 9-day storage at 4 °C [49,50]. The consistent inhibitory trend across both suspension-based and salad-based systems confirms the reliable predictive value of in vitro screening.

#### 3.2.2. Moisture Content and Weight Loss Rate

**Figure 10:**
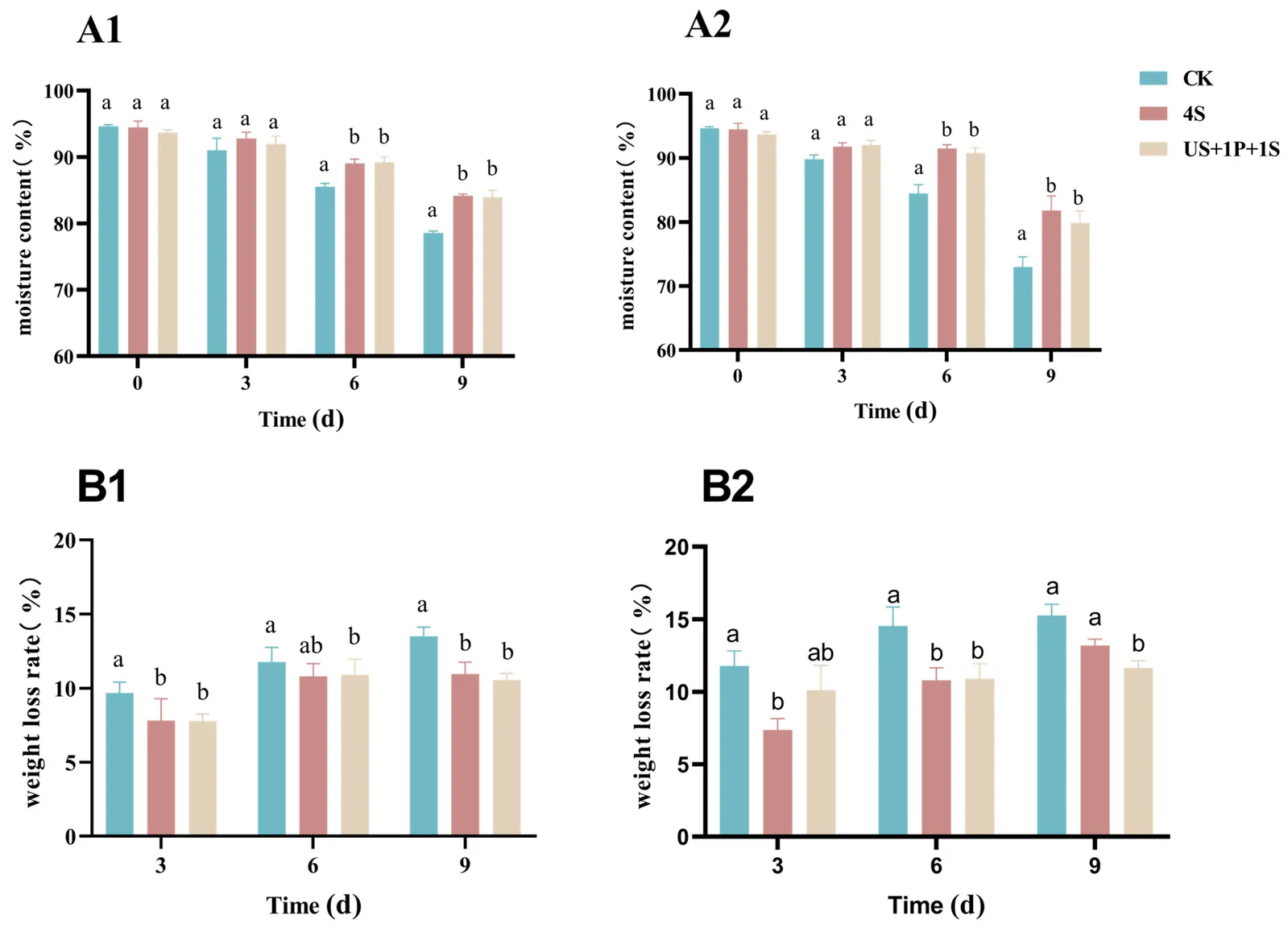
Effects of sanitization treatments on physical properties of salads. (**A1**,**A2**) Moisture content; (**B1**,**B2**) Weight loss rate. Storage conditions: (1) 4 °C; (2) 10 °C. Different lowercase letters indicate significant differences among treatments (*p* < 0.05).

US + 1P + 1S group retained higher moisture content and lower weight loss than the control group, helping maintain tissue crispness and reduce commercial dry loss during refrigerated storage and distribution (Figure 10B). The higher moisture retention in sanitized groups stems from inhibited microbial proliferation and delayed tissue senescence, which reduce metabolic water loss and cell structure breakdown [33]. This effect preserves salad crispness, slows sensory deterioration, and directly supports the extended refrigerated shelf life.

#### 3.2.3. Changes in Soluble Solids, Texture, and Color

**Figure 11:**
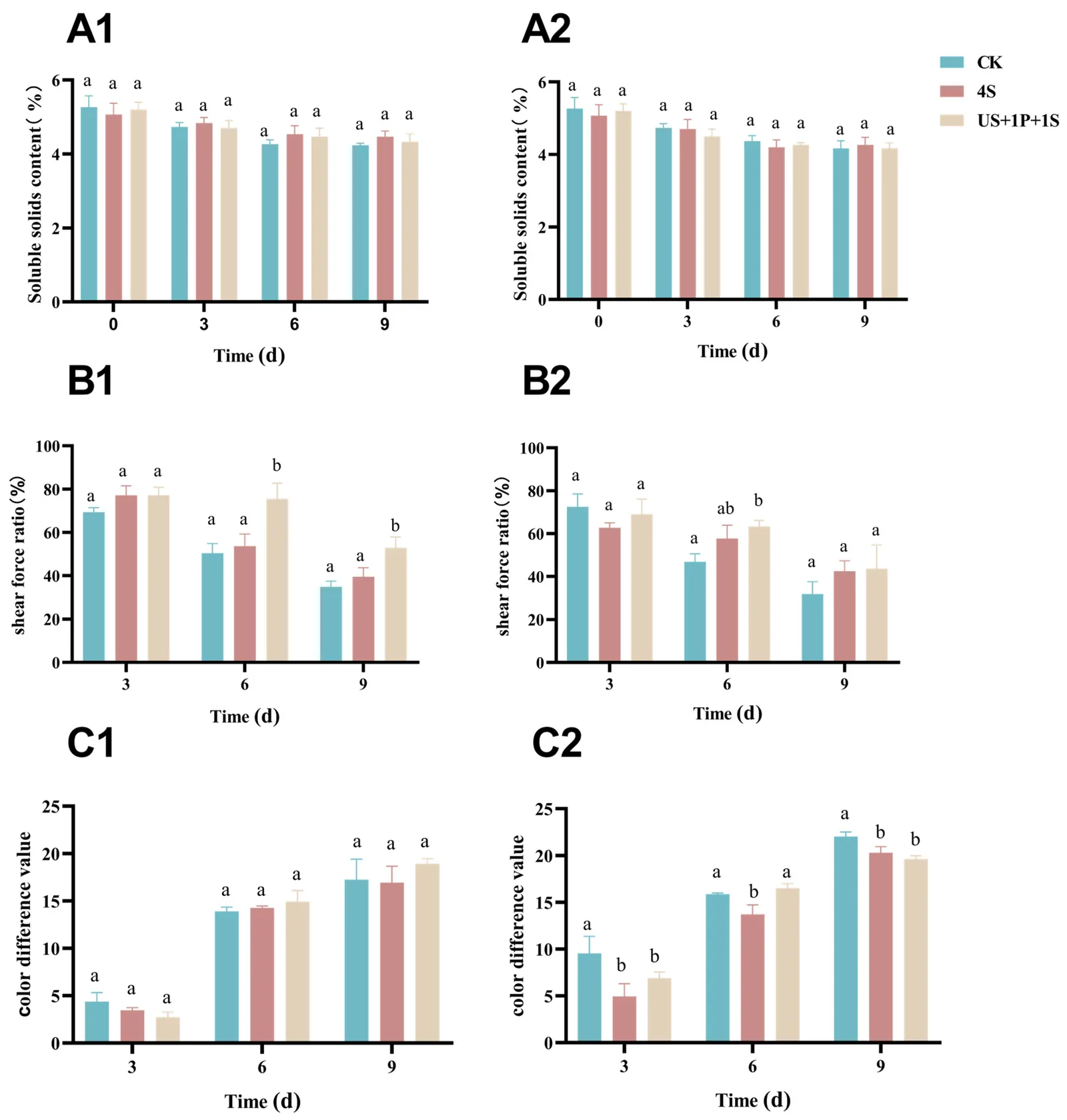
Physicochemical changes in prepared salads during storage. (**A1**,**A2**) Soluble solids content; (**B1**,**B2**) Texture (shear force ratio); (**C1**,**C2**) Color difference (ΔE). Storage conditions: (1) 4 °C; (2) 10 °C. Different lowercase letters indicate significant differences among treatments (*p* < 0.05).

Sanitization methods minimally impacted soluble solid contents at both 4 °C and 10 °C (Figure 11A). On day 6, the shear force ratio of the salad treated with the combined sanitization method was significantly higher than that of the 4S group and CK groups (Figure 11B). Furthermore, at 4 °C, the color change gradually increased with no significant variance among treatments. However, at 10 °C, the ΔE of US + 1P + 1S group was significantly lower than the control on days 3 and 9 (Figure 11C). US + 1P + 1S group preserved better firmness and less browning, effectively delaying sensory deterioration of fresh-cut vegetables. The insignificant ΔE difference across treatments at 4 °C is expected: the mild short-term sanitization causes limited cellular structure damage, restricting enzymatic browning [51]. Higher ΔE at 10 °C stems from accelerated POD activity and phenolic oxidation at warmer temperatures, consistent with faster deterioration of moisture and texture under the same condition.

**Figure 12:**
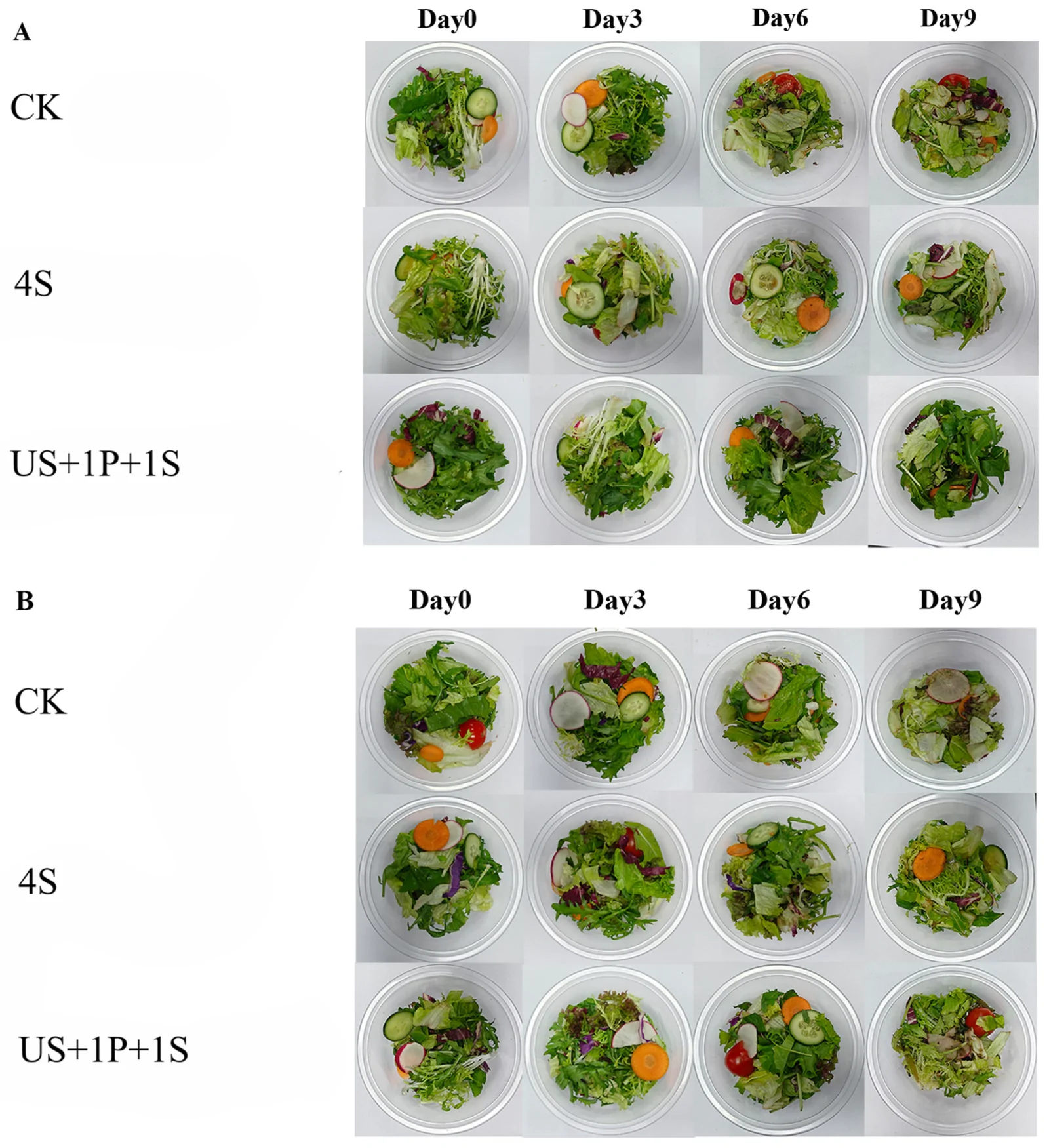
Visual appearance of prepared salads treated with different sanitization methods during storage. (**A**) Stored at 4 °C; (**B**) Stored at 10 °C.

Figure 12 presents the visual appearance of prepared salads under different treatments during 9-day storage at 4 °C and 10 °C. The combined treatment effectively retained core quality indicators including soluble solids, moisture, color and texture [51]. The higher shear force in US + 1P + 1S group reflects better firmness retention, attributed to suppressed microbial pectinase activity, inhibited endogenous cell-wall enzymes by the mildly acidic environment, and minimal tissue damage from short-term US [35,52].

These quality indicators are interlinked via a shared deterioration pathway. During storage, microbial growth produces pectinases and cellulases that degrade cell walls, leading to tissue collapse, water loss, and reduced firmness. Cell damage also releases POD and phenolic substrates, causing enzymatic browning and higher ΔE values. Combined sanitization delays this process mainly by inhibiting microbial growth, while its mild acidity and short US treatment cause minimal cellular damage. The greater quality difference at 10 °C supports this mechanism, as higher temperatures accelerate both microbial and enzymatic reactions.

#### 3.2.4. Electronic Nose Analysis

PCA of electronic nose data showed overlapping confidence ellipses for all treatments on day 0, suggesting similar initial aroma profiles [53]. However, during 4 °C storage (days 3 to 9), the ellipses completely separated, indicating that the odors of the three groups became distinguishable. At 10 °C on day 6, the 4S and CK groups still overlapped, whereas the US + 1P + 1S group was clearly separated (Figure S4).

Radar plots confirmed that the initial (day 0) odor profiles were unaffected by the sanitization methods. Subsequently, at 4 °C on day 3, the W1W sensor (sensitive to sulfides and organic sulfides) contributed most significantly to the first principal component. From days 3 to 9, the radar charts for the three groups exhibited distinct variations, aligning with the PCA results (Figure S5).

The combined treatment maintained a more stable aroma profile during storage, slowing flavor spoilage and improving consumer sensory acceptability.

#### 3.2.5. Activities of Catalase (CAT) and Peroxidase (POD) in Prepared Salads

**Figure 13:**
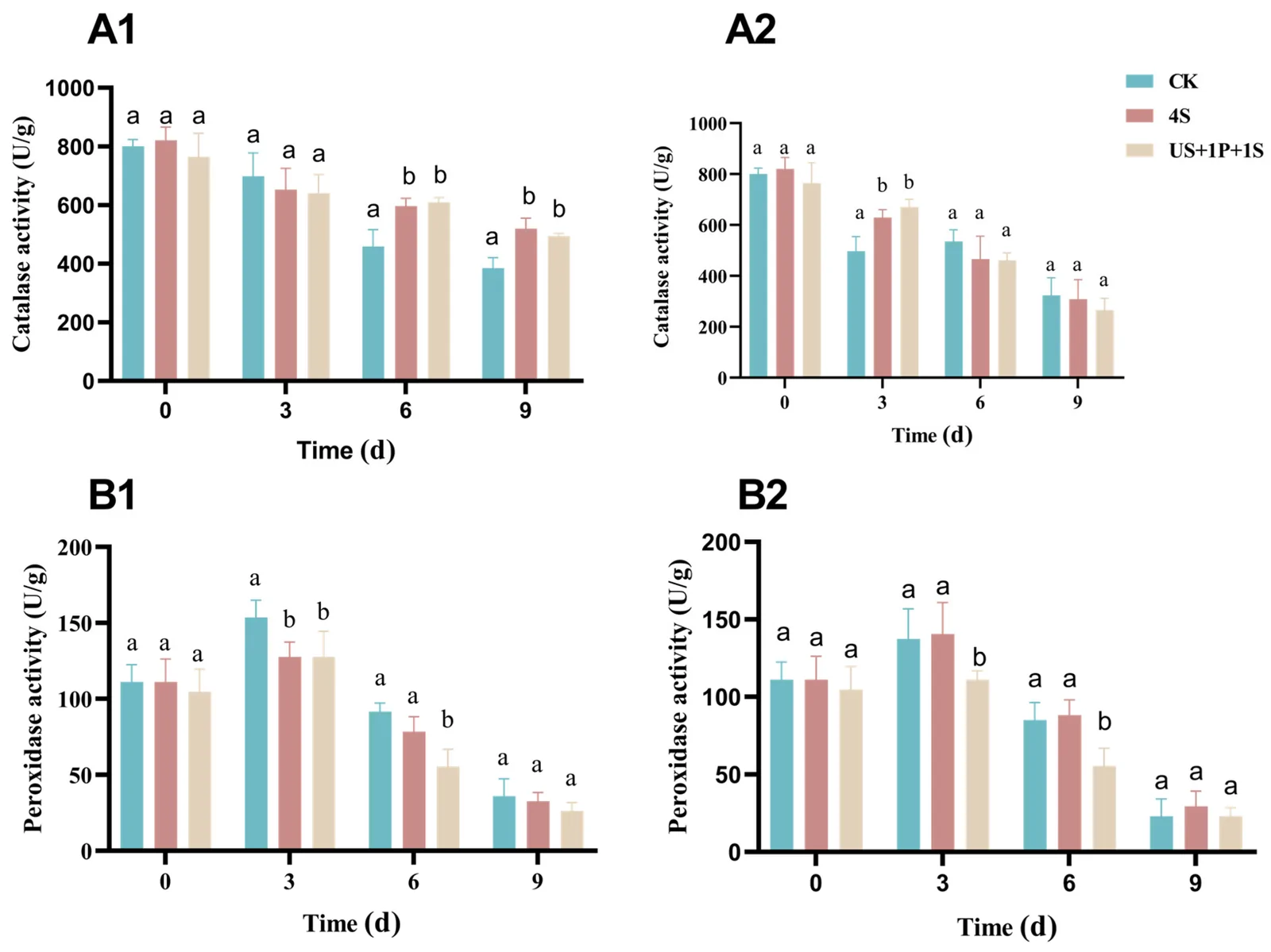
Enzyme activities in prepared salads after different sanitization treatments. (**A1**,**A2**) Catalase (CAT) activity; (**B1**,**B2**) Peroxidase (POD) activity. Storage conditions: (1) 4 °C; (2) 10 °C. Different lowercase letters indicate significant differences among treatments (*p* < 0.05).

CAT, a key antioxidant enzyme in fresh-cut produce, alleviates storage-related oxidative stress [54]. At 4 °C, CAT activity in US + 1P + 1S group was significantly higher than that of the control on day 9; at 10 °C, this significant difference appeared on day 3 and disappeared by day 6 (Figure 13A).

POD catalyzes phenol oxidation and drives enzymatic browning [55]. At both storage temperatures, POD activity in US + 1P + 1S group was significantly lower than that of the control from days 0 to day 3, followed by a gradual decrease from day 3 to day 9, which is likely attributable to oxygen depletion inside the sealed packages (Figure 13B). The inhibited POD and CAT activities are consistent with the stable ΔE values at 4 °C. These enzymes drive phenolic oxidation and enzymatic browning that are responsible for color deterioration in fresh-cut produce [52].

Preserved odor profiles and antioxidant enzyme activity help sustain consumer sensory acceptability. Meanwhile, POD suppression prolongs the refrigerated shelf life of the product. As a mild, low-dose non-thermal strategy, this combined treatment is practically implementable for industrial fresh-cut processing lines.

Traditional sanitization methods for prepared salads face significant limitations, for instance, some European countries prohibit chlorine use [48]. Although hydrogen peroxide exhibited high bactericidal effects, certain studies suggested that it might accelerate browning [55]. Conversely, the mildly acidic microenvironment (pH 5.0–6.5) maintained by SAEW and PLA likely suppressed POD activity by inducing conformational changes or providing suboptimal pH kinetics [44,55], thereby retarding enzymatic browning. This biochemical stabilization, coupled with the delayed shifts in electronic nose odor profiles, directly correlates with preserved sensory quality and retarded spoilage.

In this study, in-situ validation was conducted only for a single salad formulation. Future investigations are warranted to evaluate the efficacy of this multi-hurdle strategy in diverse fresh produce matrices. Furthermore, industrial scale-up trials and economic cost assessments are essential prior to its large-scale commercial implementation.

## 4. Conclusions

In this study, individual treatments with US, SAEW, and PLA exhibited bactericidal efficacy against *L. monocytogenes*. Notably, their combined application achieved significantly enhanced sanitization efficacy and completely inhibited the formation of VBNC cells. The synergistic treatment disrupted cell membrane integrity, as evidenced by altered membrane potential, increased permeability, and intracellular protein leakage, which ultimately resulted in bacterial death. Practical validation in prepared salad samples confirmed that the combined approach effectively reduced total plate counts and *L. monocytogenes* load, while maintaining the texture firmness, color attributes, and overall quality of salads. These findings highlight the potential of this multi-hurdle strategy for food safety control. Nevertheless, further investigations into its performance in various food matrices, industrial scalability, economic feasibility, and regulatory adaptability remain necessary prior to its large-scale commercial implementation.

## Figures and Tables

**Table 1:** Summary of all sanitization treatment groups and parameter settings.

	Treatment Group (Full Name)	Abbreviation	PLA Concentration	SAEW Concentration	Ultrasound (400 W)
Control	Untreated Control	CK	0	0	None
Single Treatments	1/4 MIC SAEW	1/4S	0	1/4 MIC	None
	1/2 MIC SAEW	1/2S	0	1/2 MIC	None
	1 MIC SAEW	1S	0	1 MIC	None
	2 MIC SAEW	2S	0	2 MIC	None
	4 MIC SAEW	4S	0	4 MIC	None
	1/4 MIC PLA	1/4P	1/4 MIC	0	None
	1/2 MIC PLA	1/2P	1/2 MIC	0	None
	1 MIC PLA	1P	1 MIC	0	None
	2 MIC PLA	2P	2 MIC	0	None
Combined Treatments	1 MIC PLA + 2 MIC SAEW	1P + 2S	1 MIC	2 MIC	None
	1 MIC PLA + 1 MIC SAEW	1P + 1S	1 MIC	1 MIC	None
	1 MIC PLA + 1/2 MIC SAEW	1P + 1/2S	1 MIC	1/2 MIC	None
	1 MIC PLA + 1/4 MIC SAEW	1P + 1/4S	1 MIC	1/4 MIC	None
	1/2 MIC PLA + 2 MIC SAEW	1/2P + 2S	1/2 MIC	2 MIC	None
	1/2 MIC PLA + 1 MIC SAEW	1/2P + 1S	1/2 MIC	1 MIC	None
	1/2 MIC PLA + 1/2 MIC SAEW	1/2P + 1/2S	1/2 MIC	1/2 MIC	None
	1/2 MIC PLA + 1/4 MIC SAEW	1/2P + 1/4S	1/2 MIC	1/4 MIC	None
Combined Treatments with Ultrasound	1 MIC PLA + 2 MIC SAEW	1P + 2S	1 MIC	2 MIC	5 min
	US + 1 MIC PLA + 1 MIC SAEW	US + 1P + 1S	1 MIC	1 MIC	5 min
	US + 1 MIC PLA + 1/2 MIC SAEW	US + 1P + 1/2S	1 MIC	1/2 MIC	5 min
	US + 1 MIC PLA + 1/4 MIC SAEW	US + 1P + 1/4S	1 MIC	1/4 MIC	5 min
	US + 1/2 MIC PLA + 2 MIC SAEW	US + 1/2P + 2S	1/2 MIC	2 MIC	5 min
	US + 1/2 MIC PLA + 1 MIC SAEW	US + 1/2P + 1S	1/2 MIC	1 MIC	5 min
	US + 1/2 MIC PLA + 1/2 MIC SAEW	US + 1/2P + 1/2S	1/2 MIC	1/2 MIC	5 min
	US + 1/2 MIC PLA + 1/4 MIC SAEW	US + 1/2P + 1/4S	1/2 MIC	1/4 MIC	5 min

Note: PLA: Phenyllactic acid; SAEW: Slightly acidic electrolyzed water; MIC: Minimum Inhibitory Concentration. The individual MIC values determined in this study were 10 mg/L for SAEW and 2.5 mg/mL for PLA. US indicates ultrasound treatment, which was applied simultaneously with the chemical sanitizers at a constant power of 400 W for 5 min.

## Data Availability

The original contributions presented in this study are included in the article/Supplementary Material. Further inquiries can be directed to the corresponding author.

## Supplementary Materials

The following supporting information can be downloaded at https://www.mdpi.com/article/10.3390/foods15183192/s1: Figures S1–S3, Tables S1 and S2, Text S1: Interpretation of the development of the PMA-qPCR method; Figures S4 and S5: Supplementary data related to electronic nose experiments; Table S3: Checkerboard assay matrix and combinations for determining the synergistic effect and Fractional Inhibitory Concentration Index (FICI) of SAEW and PLA against *Listeria monocytogenes*.

## Abbreviations

The following abbreviations are used in this manuscript:

ACC	Available Chlorine Concentration
ANS	8-Aniline-1-Naphthalenesulfonic Acid
BHI	Brain Heart Infusion
CAT	Catalase
CFU	Colony-Forming Unit
FICI	Fractional Inhibitory Concentration Index
MIC	Minimum Inhibitory Concentration
OD_600_	Optical Density at 600 nm
ONPG	ortho-Nitrophenyl-β-D-Galactopyranoside
ORP	Oxidation-Reduction Potential
PBS	Phosphate-Buffered Saline
PCA	Plate Count Agar; Principal Component Analysis
PLA	Phenyllactic Acid
PMA-qPCR	Propidium Monoazide Quantitative Polymerase Chain Reaction
POD	Peroxidase
SAEW	Slightly Acidic Electrolyzed Water
SEM	Scanning Electron Microscopy
TPA	Texture Profile Analysis
US	Ultrasound
VBNC	Viable but Non-Culturable
PI	Propidium Iodide
ANOVA	One-Way Analysis of Variance
