# Supplementary material for: Synergistic Bactericidal Efficacy of Combined Sanitization Strategies Against Listeria monocytogenes: Mechanistic Elucidation and Practical Validation in Prepared Salad
Source: Foods. 2026 Sep 9;15(18):3192. doi: 10.3390/foods15183192 (PMC13605842; doi:10.3390/foods15183192)
Supplement: Supplementary file 1 [file foods-15-03192-s001.zip › foods-4507307-supplementary.pdf]

Contents

Supplementary Figures S1–S3, Tables S1 and S2, Text S1: Interpretation of the development of the PMA-qPCR method.

Supplementary Figures S4–S5: Supplementary data related to electronic nose experiments.

Supplementary Table S3: Checkerboard assay matrix and combinations for determining the synergistic effect and Fractional Inhibitory Concentration Index (FICI) of SAEW and PLA against *Listeria monocytogenes*.

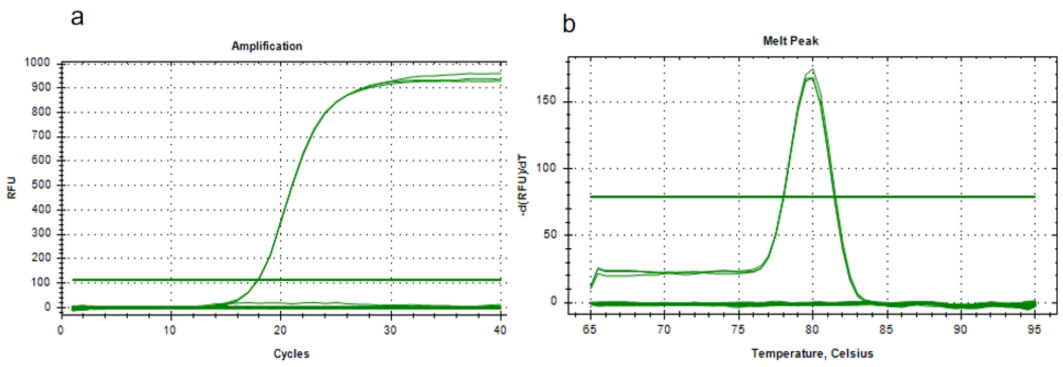

Figure S1. Specificity of the *hlyA*-based qPCR assay. (a) Amplification curves showing positive detection in *Listeria monocytogenes* reference strains and no amplification in 12 non-target strains (negative control) (Supplementary Table 1). (b) Characteristic melting peak of *L. monocytogenes* at Tm ≈ 80 °C. Results confirm high specificity of the assay and reliability of primer sign.

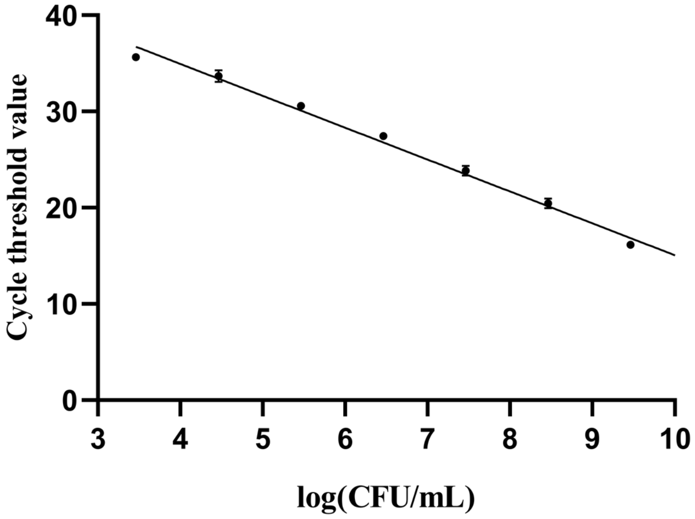

Figure S2. *Listeria monocytogenes* qPCR standard curve. Bacterial concentrations by plate counting exhibit a strong linear relationship with Ct values. Curve equation: $y = -3.314x + 48.21$ ($R^2 = 0.992$). Effective range: 1.2×10^3–1.2×10^9 CFU/mL. Enables detection of VBNC-state bacteria when combined with plate counting.

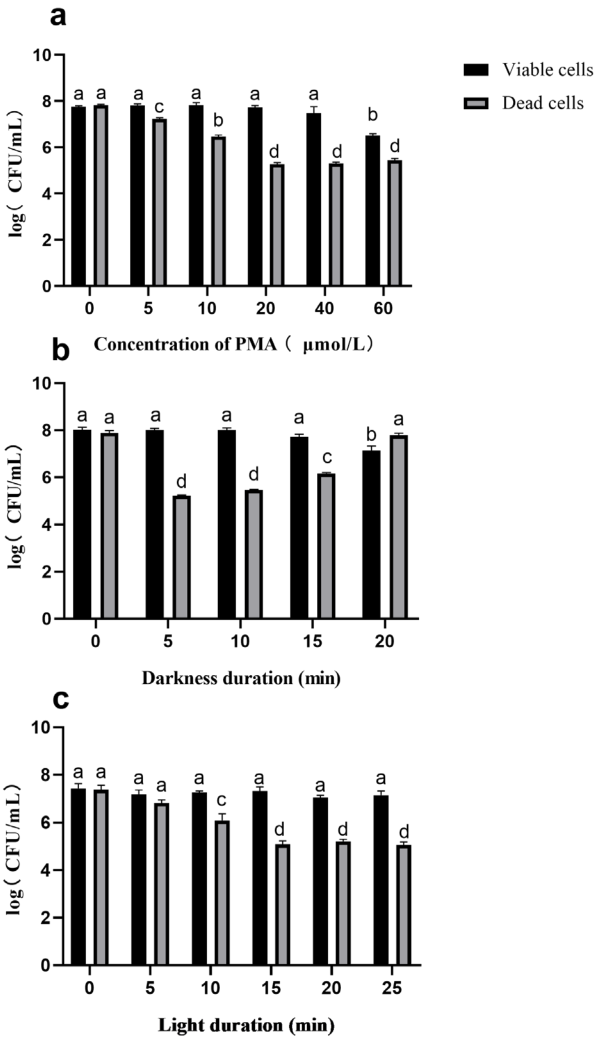

Figure S3. Effects of PMA treatment conditions on detection of viable *Listeria monocytogenes*. (a) PMA concentration (μM), (b) dark incubation time (min), (c) light exposure time (min)

Table S1. twelve non-target strains.

Scientific Name	Number
Listeria seeligeri	ATCC-35967
Listeria ivanovii	ATCC-19119
Listeria innocua	ATCC-33090
Enterococcus faecium	ATCC-29212
Staphylococcus epidermidis	CGMCC-26069

Staphylococcus aureus	ATCC-25923
Salmonella typhimurium	ATCC-14028
Escherichia coli	ATCC-25922
Shigella flexneri	ATCC-12022
Vibrio parahaemolyticus	ATCC-17802
Enterobacter aerogenes	ATCC-13048
Pseudomonas aeruginosa	ATCC-9027

Note； ATCC: American type culture collection, CGMCC: China General Microbiological Culture Collection Center.

Table S2. Detection results of qPCR and PMA-qPCR for mixed suspensions of viable and dead cells of *Listeria monocytogenes*

Live Ratio		PMA-qPCR log(CFU/mL)	qPCR log(CFU/mL)	Theoretical Live Count log(CFU/mL)	Detected Live Count log(CFU/mL)
1	-	8.33±0.10	8.47	8.47	8.33
-	1	3.28±0.09	7.02	-	3.28
100	1	8.23±0.08	8.34	8.33	8.23
10	1	8.30±0.09	8.28	8.24	8.30
1	1	8.23±0.12	8.37	8.07	8.23
1	10	7.27±0.10	8.14	7.10	7.27

Note: PMA-qPCR data represent viable counts (CFU/mL), while qPCR data indicate total bacterial loads (CFU/mL). Theoretical viable counts were calculated from total counts based on mixing ratios; detected viable counts are PMA-qPCR means. Live Ratio indicates the proportion of live cells in the mixed suspension; ‘1’ represents the sample containing 100% live cells, while ‘—’ indicates that no live cells were

detected (sample containing 100% dead cells). No significant difference between theoretical and detected values ($p > 0.05$, t-test) confirms PMA-qPCR's validity for viable-cell quantification.

Text S1. Experimental details of the PMA-qPCR assay

For the PMA-qPCR assay, PMA was added to the bacterial suspensions to a final concentration of 20 μM. The mixtures were incubated in the dark for 5 min. To cross-link the PMA to the DNA of dead cells, the samples were placed on ice and exposed to a 650 W halogen tungsten lamp at a distance of 20 cm for 15 min. Genomic DNA was then extracted. Quantitative PCR targeting the *hlyA* gene was performed in a 25 μL reaction volume containing 2 μL of DNA template, 0.2 μM of each primer (*hlyA*-F: 5'-GGTGGCTCCGCAAAAGATGAAG-3', *hlyA*-R: 5'-AGGCAATGGGAACTCCTGGTGT-3'), and 12.5 μL SYBR Green Master Mix. The thermal cycling conditions included an initial denaturation at 95 °C for 5 min, followed by 38 cycles of 95 °C for 10 s and 60 °C for 30 s.

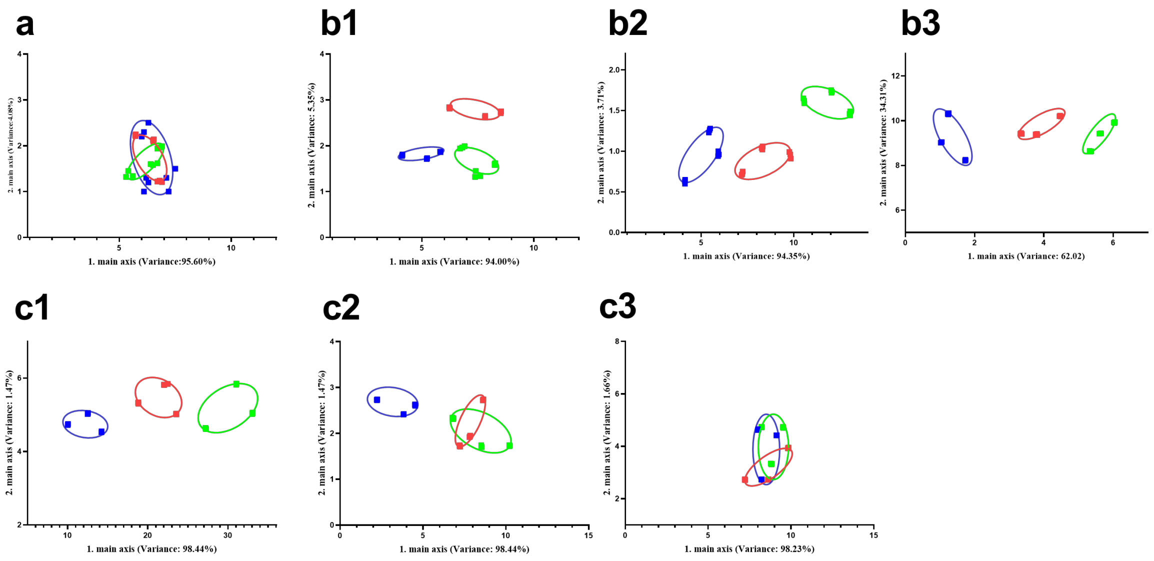

Figure S4. PCA of electronic nose analysis. (a) Day 0; (b) stored at 4 °C; (c) stored at 10 °C. Numbers 1–3 represent the 3rd, 6th, and 9th days of the storage period, respectively. Green indicates the CK group, red indicates 4S, and blue indicates the combined sanitization method (US+1P+1S).

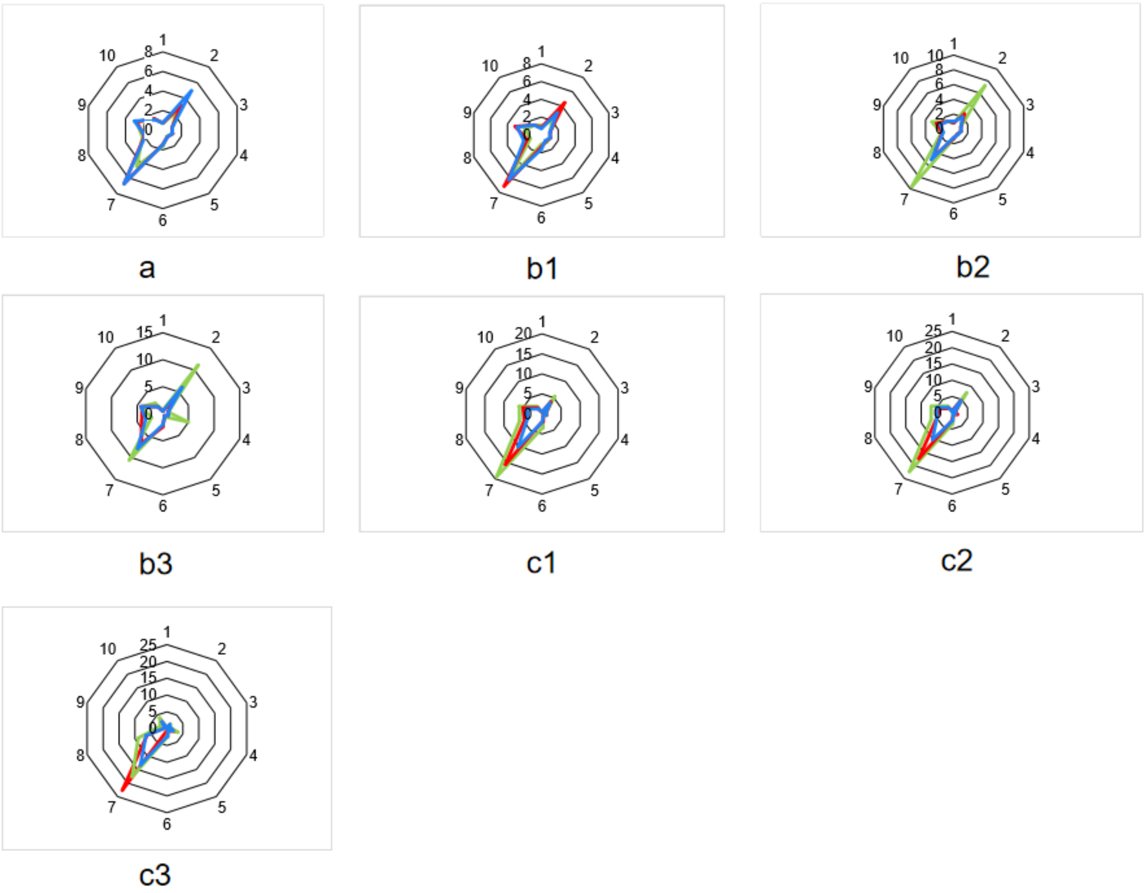

Figure S5. Radar chart of electronic nose analysis of prepared salad after treatment with different sanitization methods. (a) Day 0; (b) stored at 4 °C; (c) stored at 10 °C. Numbers 1–3 represent the 3rd, 6th, and 9th days of the storage period, respectively. Green indicates CK group, red indicates 4S, and blue indicates the combined sanitization method (US+1P+1S).

Table S3. Checkerboard assay matrix for determining the Fractional Inhibitory Concentration Index (FICI) of SAEW and PLA combinations against *Listeria monocytogenes*.

SAEW \ PLA	0	1/8 MIC	1/4 MIC	1/2 MIC	1 MIC	2 MIC	4 MIC
**0**	+	+	+	+	-	-	-
**1/8 MIC**	+	+	+	+	-	-	-
**1/4 MIC**	+	+	-	-	-	-	-
**1/2 MIC**	+	+	-	-	-	-	-

**1 MIC**	-	-	-	-	-	-	-
**2 MIC**	-	-	-	-	-	-	-
**4 MIC**	-	-	-	-	-	-	-

Notes:

(+) indicates visible bacterial growth; (-) indicates no visible bacterial growth (complete inhibition). The individual Minimum Inhibitory Concentration (MIC) values determined in this study were 10 mg/L for SAEW and 2.5 mg/mL for PLA. The bolded cell represents the optimal synergistic combination (1/4S+1/4P).
